# Assembloid CRISPR screens reveal impact of disease genes in human neurodevelopment

**DOI:** 10.1038/s41586-023-06564-w

**Published:** 2023-09-27

**Authors:** Xiangling Meng, David Yao, Kent Imaizumi, Xiaoyu Chen, Kevin W. Kelley, Noah Reis, Mayuri Vijay Thete, Arpana Arjun McKinney, Shravanti Kulkarni, Georgia Panagiotakos, Michael C. Bassik, Sergiu P. Pașca

**Affiliations:** 1 Department of Psychiatry and Behavioral Sciences, Stanford University, Stanford, CA USA; 2 Stanford Brain Organogenesis Program, Wu Tsai Neurosciences Institute and Bio-X, Stanford, CA USA; 3 Department of Genetics, Stanford University, Stanford, CA USA; 4 Eli and Edythe Broad Center of Regeneration Medicine and Stem Cell Research, University of California, San Francisco, CA USA; 5 Department of Biochemistry and Biophysics, University of California, San Francisco, CA USA; 6 Present Address: Departments of Psychiatry and Neuroscience, Black Family Stem Cell Institute, Seaver Autism Center for Research and Treatment, Alper Center for Neural Development and Regeneration, Friedman Brain Institute, Icahn School of Medicine at Mount Sinai, New York, NY USA

**Keywords:** Diseases of the nervous system, Development of the nervous system

## Abstract

The assembly of cortical circuits involves the generation and migration of interneurons from the ventral to the dorsal forebrain^1–3^, which has been challenging to study at inaccessible stages of late gestation and early postnatal human development^4^. Autism spectrum disorder and other neurodevelopmental disorders (NDDs) have been associated with abnormal cortical interneuron development^5^, but which of these NDD genes affect interneuron generation and migration, and how they mediate these effects remains unknown. We previously developed a platform to study interneuron development and migration in subpallial organoids and forebrain assembloids^6^. Here we integrate assembloids with CRISPR screening to investigate the involvement of 425 NDD genes in human interneuron development. The first screen aimed at interneuron generation revealed 13 candidate genes, including *CSDE1* and *SMAD4*. We subsequently conducted an interneuron migration screen in more than 1,000 forebrain assembloids that identified 33 candidate genes, including cytoskeleton-related genes and the endoplasmic reticulum-related gene *LNPK*. We discovered that, during interneuron migration, the endoplasmic reticulum is displaced along the leading neuronal branch before nuclear translocation. *LNPK* deletion interfered with this endoplasmic reticulum displacement and resulted in abnormal migration. These results highlight the power of this CRISPR-assembloid platform to systematically map NDD genes onto human development and reveal disease mechanisms.

## Abstract (web summary)

Assembloids are integrated with CRISPR screening to investigate the involvement of 425 neurodevelopmental disorder genes in human interneuron development, showing endoplasmic reticulum displacement before nuclear translocation and interference from *LNPK* deletion, resulting in abnormal migration.

## Main

Defects in the development and function of cortical GABAergic (γ-aminobutyric acid-releasing) interneurons have been implicated in autism spectrum disorder (ASD) and other neurodevelopmental disorders (NDDs)^5^. This is primarily based on the high prevalence of seizures and epilepsy in patients^5,7^ and neuroimaging studies that revealed changes in interneuron density and morphology in postmortem ASD tissue^8,9^. Concurrently, whole-exome sequencing and genome-wide association studies over the past decade have uncovered hundreds of ASD and other NDD-associated genes^10–13^, including several GABA receptor subunit genes related to inhibitory neural transmission^14^. It has been challenging, however, to map the role of these NDD genes onto specific stages of the protracted human interneuron development.

Cortical GABAergic interneurons are generated in the ventral forebrain and they subsequently migrate long-distances towards the dorsal forebrain to integrate into circuits^1–3^. In humans this process takes place in later stages of gestation and, unlike in rodents, continues into the postnatal period^4,15^. This protracted interneuron development is thought to contribute to increased complexity and cognitive abilities in the gyrencephalic brain^4^. To model stages of human interneuron development, we and others have developed self-organizing organoids resembling the ventral forebrain–human subpallial organoids (hSO), from human induced pluripotent stem (hiPS) cells^6,16,17^. These organoids can be integrated with human cortical organoids (hCO) to form human forebrain assembloids (hFA) and model interneuron migration and circuit assembly with glutamatergic neurons^6,16,17^. These stem cell-based three-dimensional (3D) cellular models of development and disease have either focused on studying the role of single genes in interneuron generation or migration^6,18,19^ or have leveraged screens to study the role of groups of genes in early stages of neural proliferation^20^. Mapping in parallel the impact of hundreds of NDD genes onto several stages of interneuron development, including their migration into cortical circuits at later stages of gestation, has not been achieved.

Here, we coupled human organoid and assembloid technologies with CRISPR screening to map the roles of a group of subpallium-expressed NDD genes onto interneuron generation and migration into cortical circuits. We discovered that 46 out the of 425 NDD genes (roughly 11%) interfere with interneuron development. Notably, we discovered that loss of the endoplasmic reticulum (ER)-shaping protein LNPK disrupts interneuron migration, highlighting a previously unappreciated role of ER dynamics in interneuron migration towards the cerebral cortex.

## CRISPR screens reveal NDD genes affecting interneuron development

**Extended Data Fig. 1:**
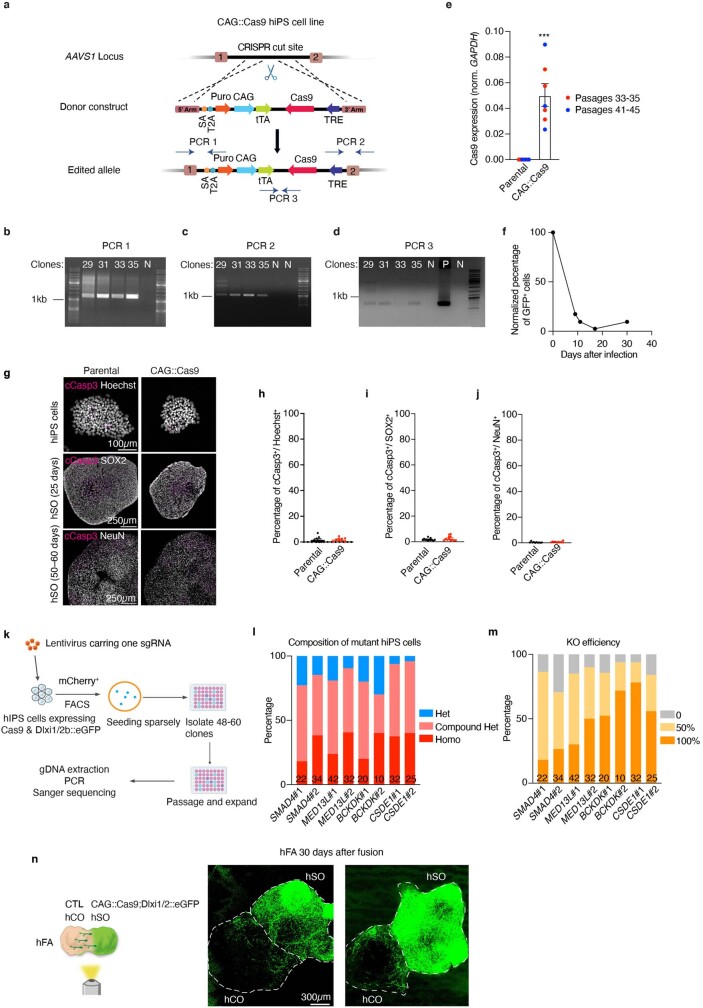
Generation and validation of the CAG::Cas9 hiPS cell line. **A**, Schematic showing the strategy to generate the CAG::Cas9 hiPS cell line. Dashed lines indicate homologous recombination sites. PCR 1–3 indicate the three pairs of primers designed to confirm the insertion of the entire donor. **b**–**d**, PCR confirming the successful amplification of the targeted DNA as shown in (**a**). “N”: negative control (PCR product from a control hiPS cell line). “P”: positive control (PCR product from the donor plasmid). In total, 35 clones were screened, and 3 clones (Clone #29, #31, and #35) were positive in all PCR reactions. **e**, Cas9 expression in CAG::Cas9 hiPS cells compared to the parental cells (n = 7 independent hiPS cell samples) by real-time-qPCR. *** P = 0.0006, two-tailed Mann-Whitney test. **f**, Genome editing efficiency in CAG::Cas9 hiPS cells. CAG::Cas9 hiPS cells were infected with a lentivirus harbouring GFP and a sgRNA against GFP. Infected cells were split into two groups with or without doxycycline (DOX) supplementation to inhibit Cas9 expression. Cells were subjected to flow cytometry to examine the percentage of GFP^+^ cells. The percentage of GFP^+^ cells (–DOX)/percentage of GFP^+^ cells ( +DOX) at each time point was plotted. **g**, Representative immunofluorescence images of hiPS cells and hSO for the quantifications in **h**–**j**. **h**–**j**, Percentage of cCasp3^+^ cells of Hoechst^+^ hiPS cells (**h**, n = 9 colonies from 3 passages) and percentage of cCasp3^+^ cells of SOX2^+^ (**i**, **n** = 12 organoids from 3 differentiations) or NeuN^+^ (**j**, n = 12 organoids from 4 differentiations) cells in hSO. **h**, **j**, Two-tailed Mann-Whitney test; **h**, P = 0.4742; **j**, P = 0.063; **i**, P = 0.1933, two-tailed unpaired t-test. **k**, Schematic showing experimental design for examining the deletion state of mutations in the CAG::Cas9;Dlxi1/2b::eGFP hiPS cells following transduction with a lentivirus expressing an individual sgRNA. **l**, Analysis of deletion state of mutant hiPS cells transduced by lentiviruses carrying 8 distinct sgRNAs. Homo: homozygous mutation. Het: heterozygous mutation. The number at the bottom of each column indicates the number of mutant clones we acquired for each sgRNA. **m**, Analysis of the KO efficiency of mutations reported in (**l**). If one allele had an in-frame deletion/insertion of less than 21 bp, its KO efficiency was assumed to be 0. **n**, Representative live images of hFA at 30 days after assembly. Day 45 CAG::Cas9;Dlxi1/2b::eGFP hSO were assembled with day 60 unlabeled hCO. The dashed lines indicate the boundary of hCO and hSO. Data are presented as mean ± s.e.m. (**e**, **h**–**j**). Source data

To determine which NDD genes interfere with cortical interneuron development, we first generated a knock-in hiPS cell line that stably expresses Cas9, which can be turned off with doxycycline (Tet-Off). To achieve this, we inserted CAG::Cas9 into the *AAVS1* locus (Extended Data Fig. 1a), generated a clonal CAG::Cas9 hiPS cell line (Extended Data Fig. 1b–d), and confirmed the expression of Cas9 (Extended Data Fig. 1e) as well as the editing efficiency by infecting the cells with a lentivirus harbouring green fluorescent protein (GFP) and a single-guide RNA (sgRNA) against GFP. Using this strategy, we observed an efficient reduction in the proportion of GFP^+^ cells within 9 days of Cas9 expression (Extended Data Fig. 1f). To assess the potential toxicity of Cas9 expression in the CAG::Cas9 line, we quantified the percentage of apoptotic cells labelled by cleaved Caspase3 (cCasp3) at many stages and found no evidence of Cas9 induced cell death (Extended Data Fig. 1g–j). To facilitate isolation of ventral forebrain interneuron lineages, we further modified the CAG::Cas9 line to stably express GFP under the control of a previously validated Dlxi1/2b enhancer that labels medial ganglionic eminence (MGE)-derived cells^6,21^. When we differentiated this CAG::Cas9;Dlxi1/2b::eGFP hiPS cell line into hSO, we detected extensive expression of GFP after 30 days of differentiation. When we assembled hSO with hCO derived from an unlabelled hiPS cell line for 30 days, we observed a notable fraction of Dlxi1/2b::eGFP^+^ cells that migrated into the cortical side of the hFA (Extended Data Fig. 1n).

**Fig. 1:**
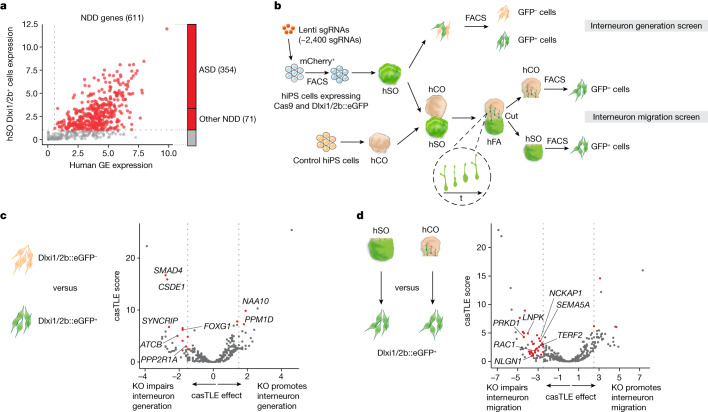
CRISPR screens of NDD genes reveal regulators of human interneuron generation and migration. **a**, A total of 425 genes (labelled in red) out of 611 NDD genes showing expression in hSO Dlxi1/2b::eGFP^+^ cells and human postconception weeks 8–9 ganglionic eminences were included in the CRISPR screens. Among these, 354 genes are associated with ASD and 71 genes are associated with other NDDs. **b**, Schematic describing the interneuron generation and migration CRISPR screens. The inset highlights how hSO-derived interneurons migrate into the hCO side of the hFA. hSO were derived from the 1205-4 hiPS cell line and hCO were derived from the 2242-1 hiPS cell line. **c**, Volcano plot of the casTLE-estimated maximum gene perturbation effect size and associated casTLE score for the interneuron generation screen. Genes with at least two sgRNAs having effects equal to or less than −1.57 or equal to or more than +1.57, roughly twice the standard deviation (s.d.) of negative controls, were selected as candidates (red), with labels for cell cycle genes and genes that were subsequently validated. **d**, Volcano plot of the casTLE-estimated maximum gene perturbation effect size and associated casTLE score for the interneuron migration screen. Genes with at least two sgRNAs having effects equal to or less than −2.5 or equal to or more than +2.5, roughly twice the s.d. of negative controls were selected as candidates (red), with labels for cytoskeleton and cell migration genes and genes that were subsequently validated.

**Extended Data Fig. 2:**
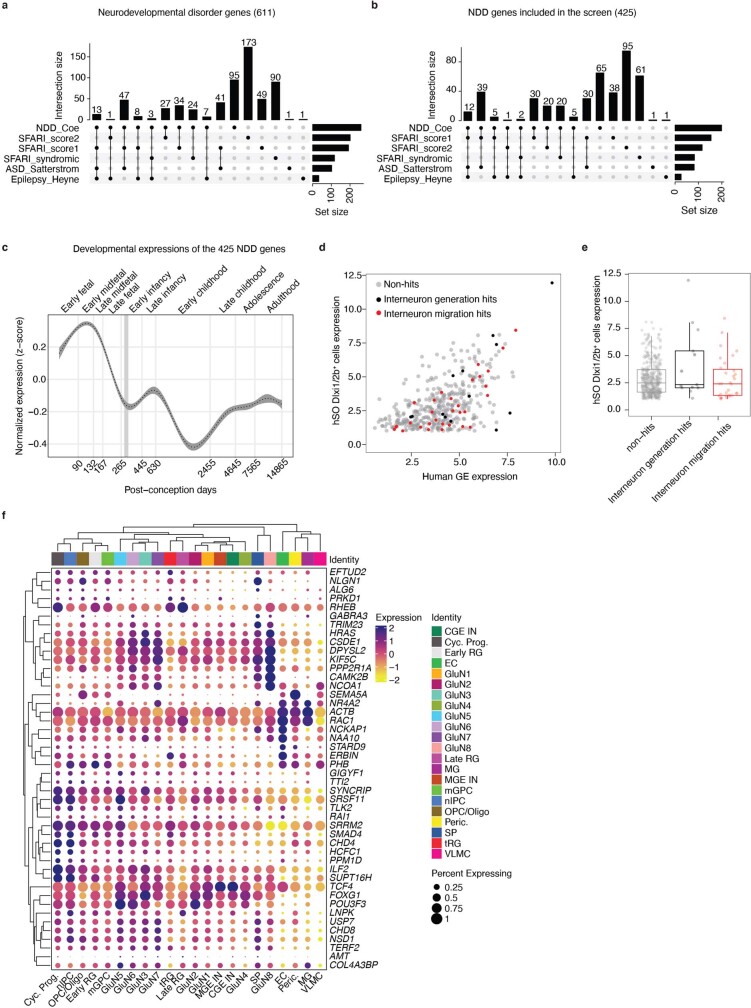
Summary of NDD genes and the genes included in the screen. **a**, Sources of the 611 NDD genes. **b**, Sources of the 425 NDD genes included in the screens. **c**, Expression trajectories in the human cerebral cortex of the selected 425 NDD genes using the BrainSpan dataset^60^. Displayed are fitted (generalized additive model; denoted with dashed line) z-score normalized expression data with 99% confidence interval shown in grey (n = 425 genes; n = 419 cortical samples). **d**, **e**, Expression in hSO Dlxi1/2b::eGFP^+^ cells of screen hit genes compared with non-hit genes. **e**, Boxplot (horizontal line denotes median; lower and upper hinges correspond to the first and third quartiles; whiskers extend 1.5 times the interquartile range with outliers shown outside this range) representation of expression levels; non-hits (n = 392 genes) versus interneuron generation hits (n = 13 genes): P = 0.2013; non-hits versus interneuron migration hits (n = 33 genes): P = 0.404; two-sided Wilcoxon tests. **f**, Expression of screen hits summarized by cell cluster based on a single-cell RNA sequencing atlas of developing human cortex^30^.

**Extended Data Fig. 3:**
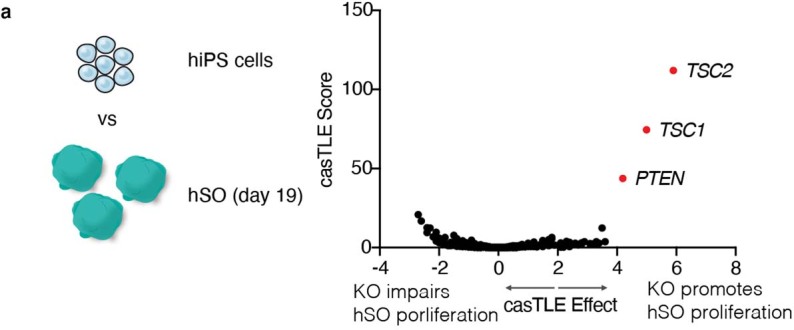
Early neural differentiation genes captured in the screen. **a**, Volcano plot of the casTLE-estimated maximum gene perturbation effect size and associated casTLE score for neural differentiation at 19 days of hSO differentiation. Three genes with notable effects are labelled in red.

To screen for genes associated with ASD and other NDDs, we compiled a list of 611 genes (Supplementary Table 1) based on genetic studies of patients with NDDs^10–13^ and the Simons Foundation Autism Research Initiative (SFARI) database (score 1, 2 and syndromic; Extended Data Fig. 2a). After excluding genes that are not expressed in hSO Dlxi1/2b::eGFP^+^ cells or human embryonic ganglionic eminences, we ended up with 425 NDD genes (Fig. 1a, 354 ASD genes and 71 genes associated with other NDDs. Extended Data Fig. 2b and Supplementary Table 2). These genes showed higher expression during prenatal than postnatal stages (Extended Data Fig. 2c), consistent with their roles in neural development. We then synthesized a sgRNA library containing five sgRNAs per candidate gene together with 13 genes with known functions in interneurons and ‘safe harbour'-targeting negative controls (218 sgRNAs), and transduced it into CAG::Cas9;Dlxi1/2b::eGFP hiPS cells (1,000 times coverage per sgRNA). The lentivirus encoding the sgRNA library also expressed mCherry, and we used fluorescence-activated cell sorting (FACS) to select transduced mCherry^+^ cells for differentiation into hSO 6 days later (Fig. 1b). To verify that our platform can detect genes that perturb early stages of neurodevelopment, such as proliferation, we performed the experiment without doxycycline and looked at proliferation related NDD genes. Indeed, sgRNAs targeting *TSC2*, *TSC1* and *PTEN*, which are known to promote neural proliferation when lost^22–24^, were enriched in hSO at 19 days of differentiation compared with hiPS cells (Extended Data Fig. 3a). We next investigated the heterozygous and/or homozygous state of clones by testing the effect of eight sgRNAs targeting four genes (two randomly selected genes from the library and two hit genes). We produced eight individual lentiviruses encoding these eight sgRNAs and infected CAG::Cas9;Dlxi1/2b::eGFP hiPS cells (Extended Data Fig. 1k). By isolating individual clones, we identified 217 mutant clones, most of which were either homozygous deletions or compound heterozygous (compound HET) deletions, with varying percentages of frame-shift deletions (Extended Data Fig. 1l,m).

To identify genes that affect interneuron generation, we collected hSO at 44 days of differentiation and used FACS to separate the Dlxi1/2b::eGFP^+^ cells (Fig. 1b). We evaluated the relative enrichment of sgRNAs for each targeted gene when comparing the eGFP^+^ and eGFP^–^ populations, and we identified 13 genes whose sgRNAs have concordant effects that significantly deviate from the negative control sgRNA distribution (Fig. 1c and Supplementary Table 3). Unlike cell viability screens that directly compare sgRNAs enrichment in the initial and final populations^25^, we compared proportional enrichment of sgRNAs in cell populations of interest at the end of the screen. Therefore, sgRNAs targeting genes with replication and survival effects before interneuron specification should be equally enriched in the eGFP^+^ and eGFP^−^ cell populations, which prevented us from identifying those genes as hits for the interneuron generation screen. The candidate genes for interneuron generation included five cell cycle-related genes, including *PPP2R1A*, *PPM1D*, as well as *FOXG1*, which is critical for forebrain interneuron development^26^ and the loss of which results in NDDs^27^.

To prioritize ASD and NDD genes that regulate interneuron migration, we next created hFA by integrating unperturbed hiPS cell-derived hCO at day 60 and CRISPR-perturbed hiPS cell-derived hSO at 45 days of differentiation (Fig. 1b). We generated more than 1,000 hFA to achieve an estimated coverage of 500 migrated cells per sgRNA. We allowed interneurons from hSO to migrate into hCO for 30 days; we then separated the two sides of the hFA and used FACS to isolate Dlxi1/2b::eGFP^+^ cells from the hSO part and migrated Dlxi1/2b::eGFP^+^ cells from the hCO part (Fig. 1b). We evaluated the relative enrichment of sgRNAs for each targeted gene in migrated and non-migrated Dlxi1/2b::eGFP^+^ cells. Similar to the strategy used in the interneuron generation screen, comparing proportional enrichment of sgRNAs in migrated and non-migrated Dlxi1/2b::eGFP^+^ cells prevented us from identifying genes regulating survival or proliferation before migration as false positive hits. This enabled us to reliably detect genes with specific roles in interneuron migration. Our analysis identified 33 candidate genes affecting interneuron migration (Fig. 1d and Supplementary Table 4). Five of these genes were related to cytoskeleton organization and cell migration, including *NCKAP1*, *SEMA5A* and *PRKD1* as well as *RAC1*, a main regulator of cytoskeleton dynamics^28^. This is consistent with previous studies suggesting that functional repression of *Rac1* in mice impairs neuronal migration by affecting the leading process of migrating interneurons^29^. We note that the candidate genes identified in the screen show similar expression levels as non-hit genes, indicating that these screens can capture effects from both highly and lowly expressed genes (Extended Data Fig. 2d,e). When we plotted the expression of hit genes on an atlas of developing human cortex^30^, we observed broad expression across many cell types (Extended Data Fig. 2f). These findings suggest that mutation of these genes could result in dysfunction of interneurons as well as other cell types.

## *SMAD4* and *CSDE1* loss impairs subpallium differentiation

**Fig. 2:**
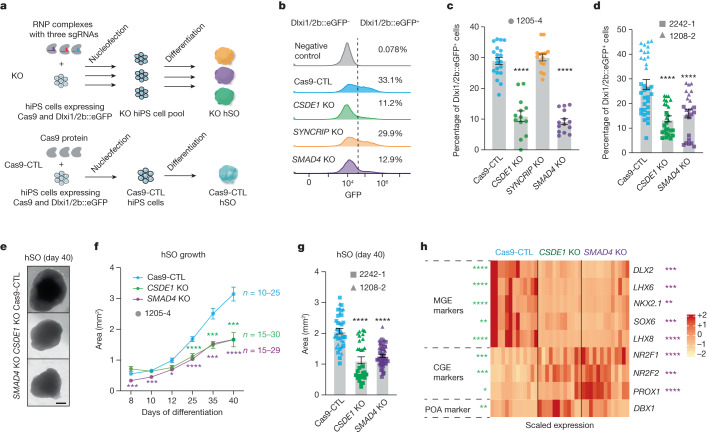
*CSDE1* and *SMAD4* regulate human interneuron generation. **a**, Schematic showing the generation of KO cell pools. Premixed Cas9 protein and sgRNAs (three sgRNAs per gene, ribonucleoprotein (RNP) complex) were nucleofected into CAG::Cas9;Dlxi1/2b::eGFP hiPS cells, followed by differentiation and flow cytometry analysis. Cas9-CTL, cells received Cas9 protein alone. **b**, Percentages of Dlxi1/2b::GFP^+^ cells by flow cytometry analysis in day 45 hSO. Negative control: hSO without GFP expression. The *x* axis shows GFP intensity and the *y* axis shows counts. **c**,**d**, Percentage of Dlxi1/2b::GFP^+^ cells in hSO engineered from hiPS cell line 1205-4 (**c**, day 45 hSO, Cas9-CTL, *n* = 20, *CSDE1* KO, *n* = 13, *SYNCRIP1* KO, *n* = 14 and *SMAD4* KO, *n* = 14, from two nucleofections and differentiations) or from 2242-1 and 1208-2 hiPS cell lines (**d**, day 40 hSO Cas9-CTL, *n* = 34; *CSDE1* KO, *n* = 27 and *SMAD4* KO, *n* = 25, from two differentiations). Each organoid was processed as one sample. **e**, Bright field images of hSO. Scale bar, 400 µm. **f**, Area of hSO (from four differentiations). **g**, Comparison of day 40 hSO size derived from 2242-1 and 1208-2 hiPS cell lines (Cas9-CTL, *n* = 37; *CSDE1* KO, *n* = 30 and *SMAD4* KO, *n* = 47, from two differentiations). **h**, Heatmap showing the differential expression of transcription factors in 35–40 days Cas9-CTL and KO hSO examined by RT–qPCR (*n* = 12–13, from seven differentiations and three hiPS cell lines); two-tailed Mann–Whitney test with Benjamini–Hochberg adjusted *P* value. Stars represent comparisons between Cas9-CTL and *CSDE1* (left) or *SMAD4* (right) KO. Data are presented as mean ± s.e.m. (standard error of mean, **c**,**d**,**f**,**g**); one-way ANOVA (**c**, *F*_3,57_ = 78.64; **d**, *F*_2,83_ = 20.70; **g**, *F*_2,111_ = 38.74) or mixed model two-way ANOVA (**f**, genotype as factor, *F*_2,81_ = 45.29) using Dunnett’s multiple comparison test. ****P < 0.0001. Supplementary Table 5 shows sample size and *P* values for **f** and **h**. Source data

**Extended Data Fig. 4:**
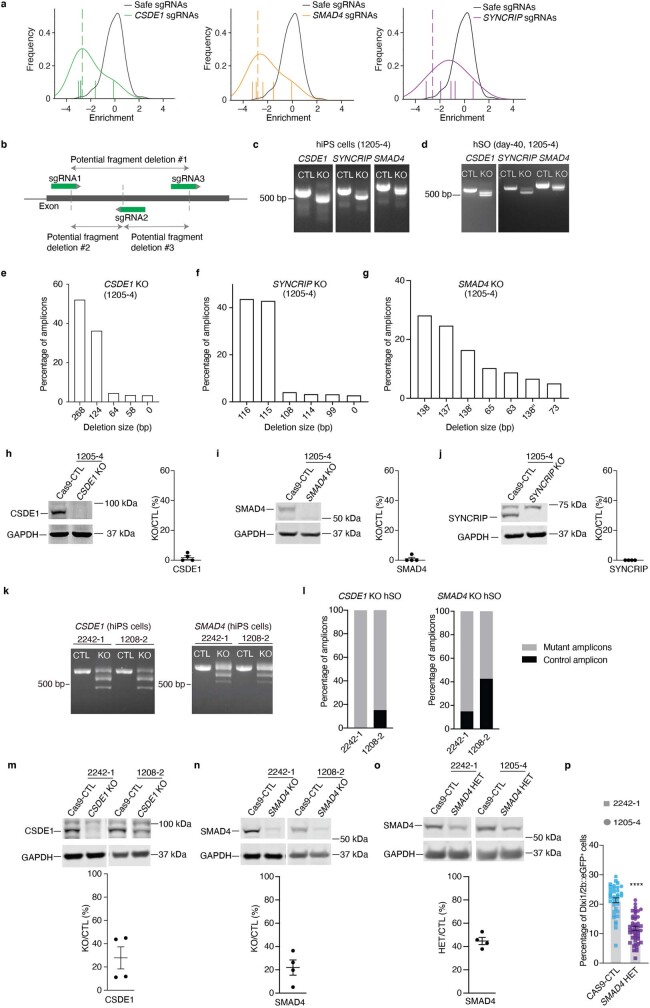
Confirmation of the KO efficiency for the selected candidate genes: *CSDE1* and *SMAD4*. **a**, Estimation of gene perturbation effect sizes. Log_2_ fold change enrichments, normalized to the median of negative ‘safe’-targeting controls, of sgRNAs between the Dlxi1/2::eGFP^+^ and Dlxi1/2::eGFP^−^ samples are plotted in the indicated color; the distribution of safe-targeting sgRNA controls in black. The dashed line is the casTLE-estimated maximum effect size. **b**, Schematic showing 3 sgRNAs designed to target an exon of the gene of interest to generate multiple fragment deletions. **c**–**d**, Genotype analysis of hiPS cells (**c**) or day–40 hSO (**d**) showing the PCR amplicon of individual candidate genes. These experiments have been repeated 3 times. **e**–**g**, Amplicon sequencing results from genomic DNA extracted from day–40 hSO derived from *CSDE1* KO (**e**), *SYNCRIP* KO (**f**) or *SMAD4* KO (**g**) cells. The percentage of each amplicon that is more than 2% is presented. In (**g**), 138, 138’ and 138” indicate three different amplicons that showed a 138 bp deletion. **h**–**j**, Representative western blotting images and quantifications for KO cell pools generated from the 1205-4 hiPS cell line (**h**, **i**, n = 4, ~day 40 hSO, 2 differentiations; **j**, n = 4, hiPS cells). GAPDH was used as a loading control. **k**, Genotype analysis of hiPS cells showing the PCR amplicon of *CSDE1* or *SMAD4*. These experiments have been repeated 3 times. **l**, Amplicon sequencing results from genomic DNA extracted from day 40 hSO. **m**–**o**, Representative western blotting images and quantification of CSDE1 (**m**) and SMAD4 (**n**, **o**) abundance in day 40 hSO (n = 4 differentiations for each). GAPDH was used as a loading control. **p**, Percentage of Dlxi1/2b::GFP^+^ cells in ~day–40 hSO (Cas9-CTL, n = 37, *SMAD4* HET, n = 46, from 3 differentiations). Two-tailed Mann-Whitney test. **** P < 0.0001. Data are presented as mean ± s.e.m. (**h**–**j**, **m**–**p**). Source data

To confirm the role of the candidate genes in subpallium development, we decided to validate the top hits from the interneuron generation screen: *SMAD4*, *CSDE1* and *SYNCRIP* (Fig. 1c and Extended Data Fig. 4a). We engineered the CAG::Cas9;Dlxi1/2b::eGFP hiPS cell line by nucleofecting ribonucleoprotein complexes composed of Cas9 protein and three sgRNAs per gene targeting each of these gene hits and generated knock-out (KO) hiPS cell pools for each candidate (Fig. 2a and Extended Data Fig. 4b). Both hiPS cells and hSO at 40 days of differentiation showed efficient deletion of the corresponding genes (Extended Data Fig. 4c,d). We found that KO cell pool-derived hSO at 40 days of differentiation contained more than 96% of sequenced amplicons with partial deletion (Extended Data Fig. 4e–g) and showed very little protein expression of the mutated gene (Extended Data Fig. 4h–j). At 45 days of differentiation, we examined hSO by flow cytometry and found that *SMAD4* and *CSDE1* KO hSO (****P < 0.0001), but not *SYNCRIP* KO hSO (*P* = 0.87), generated reduced proportions of Dlxi1/2b::eGFP^+^ cells compared with the Cas9-control (Cas9-CTL, Fig. 2b,c). To further investigate this phenotype, we generated *SMAD4* and *CSDE1* KO cells from two more hiPS cell lines that stably express eGFP under the control of the Dlxi1/2b enhancer (Extended Data Fig. 4k–n). We observed a similar reduction in the percentage of Dlxi1/2b::eGFP^+^ cells in hSO derived from KO hiPS cells (Fig. 2d, ****P < 0.0001). Moreover, we generated *SMAD4* HET KO lines as mutations in patients are generally heterozygous (Extended Data Fig. 4o). We found that *SMAD4* HET lines also showed impaired interneuron generation (Extended Data Fig. 4p). Furthermore, we found that *SMAD4* and *CSDE1* KO hSO were consistently smaller in size than Cas9-CTL at many in vitro differentiation stages (Fig. 2e–g, *P* < 0.01). Several studies have suggested that both *SMAD4* and *CSDE1* are involved in neural differentiation^31,32^, so we next examined the expression of transcription factors that regulate subpallium development. Consistent with the reduced proportion of Dlxi1/2b::eGFP^+^ cells, we detected a strong reduction of *DLX2* expression in both *SMAD4* and *CSDE1* KO hSO at 35–40 days of differentiation (Fig. 2h, ****P < 0.0001, ***P = 0.0009). In addition, *SMAD4* and *CSDE1* KO hSO showed a switch in ventral forebrain identity with reduced expression of several MGE-expressed transcription factors (*NXK2.1*, *LHX6*, *LHX8* and *SOX6*, refs. ^33,34^) and increased expression of caudal ganglionic eminence-related transcription factors (*NR2F2*, *NR2F1* and *PROX1*, refs. ^33,34^) (Fig. 2h, *P* < 0.03). We also observed that *CSDE1* KO hSO showed increased expression of the transcription factor *DBX1*, which plays a critical role in the development of the preoptic area^35^. Altogether, these results demonstrated that deletion of *SMAD4* and *CSDE1* reduces the proportion of Dlxi1/2::eGFP^+^ lineage cells in hSO, likely resulting from disrupted subpallium patterning.

## *TERF2* and *LNPK* loss impairs interneuron migration

**Fig. 3:**
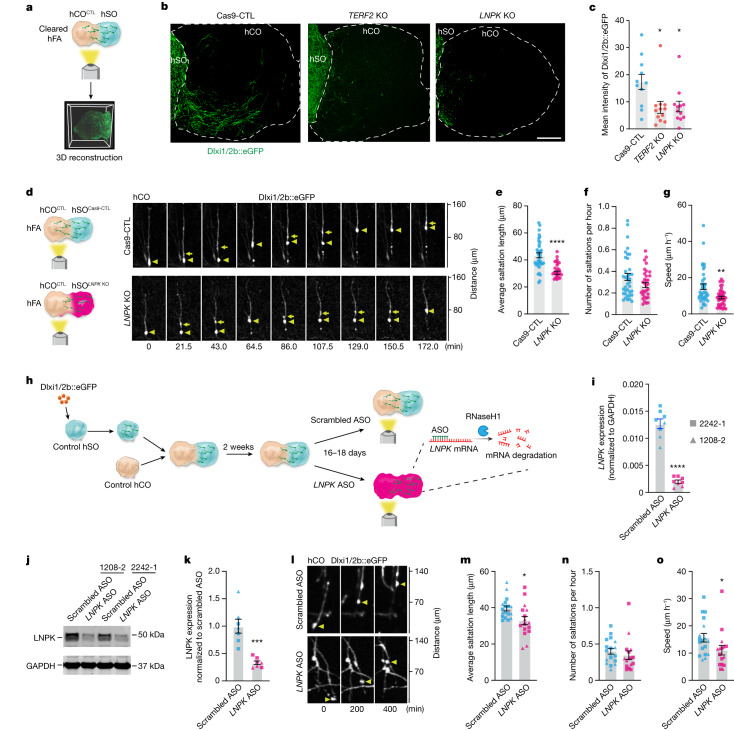
Deletion of *TERF2* and *LNPK* impairs interneuron migration in hFA. **a**, Schematic showing CUBIC-cleared hFA imaging and 3D reconstruction. **b**, Representative images of 2D projections from reconstructed 3D images of hFA. Scale bar, 400 μm. **c**, Mean intensity of GFP (Cas9-CTL, *n* = 11 hFA; *TERF2* KO, *n* = 12 hFA and *LNPK* KO, *n* = 12 hFA, from two differentiations). Cas9-CTL versus *TERF2* KO: *P = 0.0225; Cas9-CTL versus *LNPK* KO: *P = 0.0428, Kruskal–Wallis test with Dunn’s multiple comparisons test. **d**, Saltatory migration of Dlxi1/2b::eGFP^+^ cells in the hCO side of hFA. **e**–**g**, Saltation length (**e**), number of saltations (**f**) and speed of movement (**g**) of Dlxi1/2b::eGFP^+^ cells (*n* = 38 cells for each from 11 Cas9-CTL and 13 *LNPK* KO hFA from four differentiations). Two-tailed unpaired *t*-test, ****P *<* 0.0001 (**e**). Two-tailed Mann–Whitney test, *P* = 0.1332 (**f**) and **P = 0.0021 (**g**). **h**, The experimental design using an ASO to acutely delete LNPK and observe the effects on interneuron migration. **i**, Quantification of *LNPK* mRNA. ****P < 0.0001, two-tailed unpaired *t*-test (*n* = 8 hFA from three differentiations). **j**,**k**, Representative western blotting images (**j**) or analysis (**k**) (*n* = 8 hFA from three differentiations). ***P = 0.0006, two-tailed Mann–Whitney test. **l**, Representative time-lapse images showing saltatory migration of Dlxi1/2b::eGFP^+^ cells. **m**–**o**, Saltation length (**m**), number of saltations (**n**) and speed of movement (**o**) of Dlxi1/2b::eGFP^+^ cells (scrambled ASO, *n* = 18 cells from nine hFA; *LNPK* ASO, *n* = 16 cells from eight hFA. Each from four differentiations of two hiPS cell lines). Two-tailed unpaired *t*-test, *P = 0.0109 (**m**). Two-tailed Mann–Whitney test, *P* = 0.2037 (**n**) and *P = 0.0229 (**o**). Data are presented as mean ± s.e.m (**c**,**e**–**g**,**i**,**k**,**m**–**o**). Source data

**Extended Data Fig. 5:**
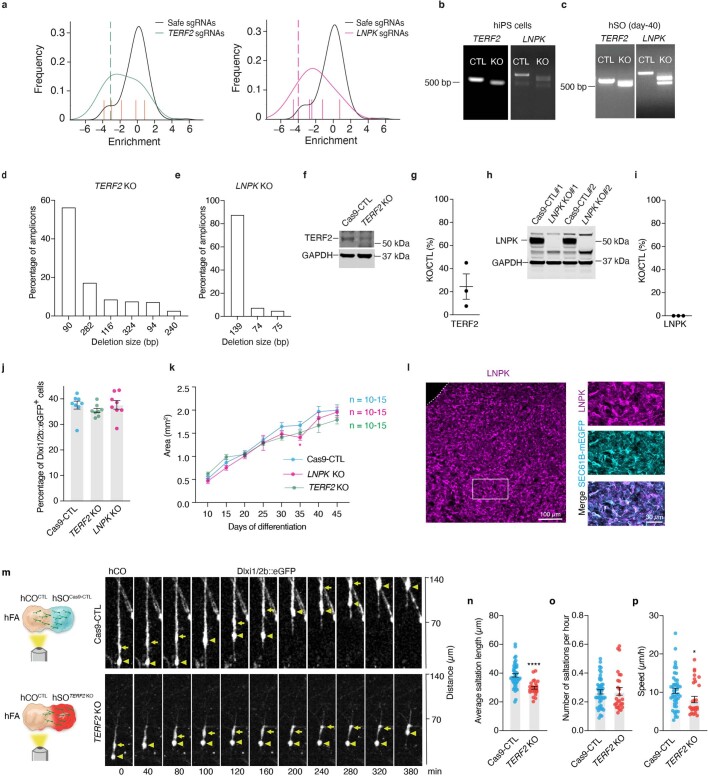
Confirmation of KO efficiency of *TERF2* and *LNPK* and effects of *TERF2* loss on interneuron saltatory migration. **a**, Estimation of gene perturbation effect sizes. Log_2_ fold change enrichments, normalized to the median of negative ‘safe’-targeting controls, of sgRNAs between the Dlxi1/2::eGFP^+^ and Dlxi1/2::eGFP^−^ samples are plotted in the indicated color; the distribution of safe-targeting sgRNA controls is shown in black. The dashed line is the casTLE-estimated maximum effect size. **b**–**c**, Genotype analysis of hiPS cells (**b**) or day 40 hSO (**c**) showing the PCR amplicon of individual candidate genes. These experiments have been repeated 3 times. **d**–**e**, NGS analysis of amplicons amplified from genomic DNA extracted from day 40 hSO derived from *TERF2* KO (**d**) and *LNPK* KO (**e**) cells. The percentage of each amplicon that is more than 2% is presented. In (**d**), *TERF2* 116’ has an additional 21 bp insertion. **f**–**i**, Representative western blotting images and quantifications showing TERF2 (**f**, **g**) and LNPK (**h**, **i**) abundance in KO and Cas9-CTL hSO (day 60–80, n = 3 samples). GAPDH was used as a loading control. **j**, Percentage of Dlxi1/2b::GFP^+^ cells in day–57 hSO (n = 8 from 2 differentiations). Each organoid was processed as one sample. Kruskal-Wallis test (P = 0.1316). **k**, hSO size over time (from 4 differentiations). mixed model two-way ANOVA (genotype as factor, F_2,42_ = 3.329) using Dunnett’s multiple comparison test. * P = 0.0324. **l**, Left, a representative immunofluorescence image showing LNPK expression in hSO. Right, inset showing LNPK co-localizes with SEC61B-mEGFP in hSO derived from SEC61B-mEGFP hiPS cells. This experiment has been repeated 3 times. **m**, Saltatory migration of Dlxi1/2b::eGFP^+^ cells in the hCO side of the Cas9-CTL and *TERF2* KO hFA. Triangles mark the nucleus and arrows mark the dilation that moves before nuclear translocation. **n**–**p**, Saltation length, number of saltations, and speed of movement of Dlxi1/2b::eGFP^+^ cells in the hCO side of the hFA (Cas9-CTL, n = 47 cells from 14 hFA; *TERF2* KO, n = 26 cells from 8 hFA, from 3-4 differentiations). **n**, **** P < 0.0001, two-tailed unpaired t-test. **o**, **p**, Two-tailed Mann-Whitney test. **o**, P = 0.5497, **p**, * P = 0.0267. Data are presented as mean ± s.e.m. (**g**, **i**, **j**, **k**, **n**–**p**). Source data

We next inspected the list of NDD gene hits for interneuron migration screen with a focus on genes that have not been generally associated with cell migration. We found *TERF2*, a component of the shelterin complex that regulates telomere length^36^, and *LNPK*, which is proposed to serve as a curvature-stabilizing protein within tubular three-way junctions of the ER^37^ (Fig. 1d and Extended Data Fig. 5a). To verify whether these genes modulate interneuron migration, we engineered the CAG::Cas9;Dlxi1/2b::eGFP hiPS cells to generate KO cell pools for both *TERF2* and *LNPK* and confirmed the deletion of both genes (Extended Data Fig. 5b–i). Unlike hits from the first screen that affect interneuron generation, the loss of *TERF2* or *LNPK* did not affect the proportions of Dlxi1/2b::eGFP^+^ cells in hSO or organoid size over time (Extended Data Fig. 5j,k), suggesting that they do not affect interneuron generation. We then measured fluorescence intensity of Dlxi1/2b::eGFP in unlabelled hCO fused with KO or Cas9-CTL hSO to estimate cellular migration (Fig. 3a and Supplementary Video 1). Compared to Cas9-CTL hFA, we observed reduced mean intensity of GFP in the hCO assembled with the *TERF2* or *LNPK* KO hSO (Fig. 3b,c, Cas9-CTL versus *TERF2* KO: *P = 0.0225; Cas9-CTL versus *LNPK* KO: *P = 0.0428). This suggests that loss of *TERF2* or *LNPK* led to reduced migration of Dlxi1/2b::eGFP^+^ cells in forebrain assembloids.

## Loss of LNPK results in abnormal interneuron migration

Homozygous loss-of-function mutations in *LNPK* have been reported in patients with severe epilepsy^38^, which could involve abnormal development of cortical interneurons. To investigate the role of LNPK in interneuron migration into the cerebral cortex, we performed live imaging to examine in detail the movement of *LNPK* KO interneurons in hFA. Saltatory migration of interneurons involves, at the cellular level, two phases: first, the centrosome and the Golgi apparatus migrate forwards in the leading branch and form a swelling; then, the nucleus translocates towards the swelling^39–41^. We monitored Dlxi1/2b::eGFP^+^ cells in the hCO side of hFA generated by fusing unlabelled hCO with Cas9-CTL or *LNPK* KO hSO, and we discovered reduced saltation length and reduced speed of movement in *LNPK* KO migratory eGFP^+^ cells compared with Cas9-CTL cells (Fig. 3d–g, ****P < 0.0001, **P = 0.0021 and Supplementary Video 2). We observed a similar defect in the *TERF2* KO cells (Extended Data Fig. 5m–p, ****P < 0.0001; *P = 0.0267). This indicates that loss of *LNPK* or *TERF2* results in a migration defect by primarily affecting the saltation length of Dlxi1/2b::eGFP^+^ cells, which depends on calcium-dependent cytoskeletal changes, as previously shown^18,41^. To determine whether LNPK acutely contributes to interneuron migration or whether the impaired migration is due to developmental defects, we also used an antisense oligonucleotide (ASO) targeting *LNPK* mRNA. We labelled interneuron lineages in hSO with a Dlxi1/2b::eGFP reporter. We then assembled hFA from both control hCO and hSO for 14 days, and directly applied *LNPK* ASO or a scrambled control for another 16–18 days (Fig. 3h). We confirmed that *LNPK* ASO treatment reduced *LNPK* mRNA amounts by 85% and protein amounts by 70% (Fig. 3i–k, ****P < 0.0001; ***P = 0.0006). As with our Cas9 engineered lines, we observed reduced saltation length and slower speed of movement in the *LNPK* ASO-treated cells (Fig. 3l–o, *P < 0.02). These findings indicate that LNPK is critical for saltatory migration of human interneurons.

**Extended Data Fig. 6:**
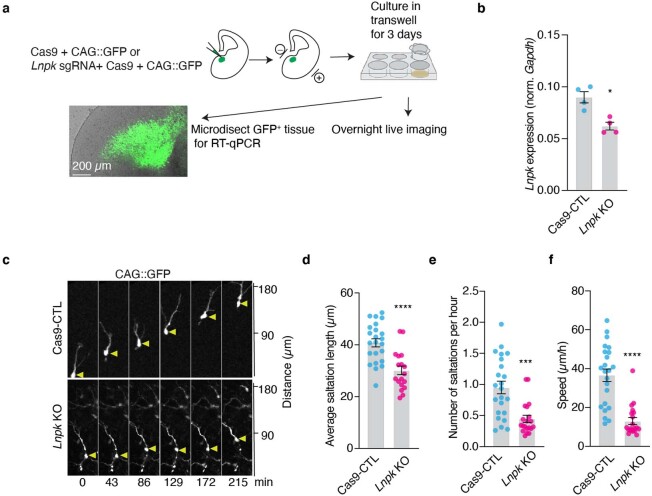
Loss of *Lnpk* affects mouse interneuron migration. **a**, Schematic illustrating the experimental design for investigating the role of LNPK in mouse interneuron migration. **b**, Quantification of *Lnpk* mRNA expression in the microdissected tissue (n = 4, each datapoint represents microdissected slices from 2-3 embryos). Data are presented as mean ± s.e.m.; two-tailed Mann-Whitney test. * P = 0.0286. **c**, Representative time-lapse sequences of live cell imaging showing the saltatory migration of GFP^+^ cells in slices electroporated with pCAG-IRES-GFP and CAS9 protein or pre-mixed sgRNAs-Cas9 complex targeting mouse *Lnpk*. Triangles mark the nucleus. **d**–**f**, Quantification of the saltation length (**d**, two-tailed unpaired t-test; **** P < 0.0001), number of saltations (**e**, two-tailed Mann-Whitney test; *** P = 0.0002), and speed of movement of electroporated cells (**f**, two-tailed Mann-Whitney test; **** P < 0.0001). Data are presented as mean ± s.e.m.; Cas9-CTL, n = 23 cells; *Lnpk* KO, n = 20 cells; from 10 embryos). Source data

To further validate our findings, we investigated the role of LNPK in mouse interneuron migration. Specifically, we coelectroporated a CAG-IRES-GFP plasmid with either a premixed sgRNAs–Cas9 complex targeting mouse *Lnpk* (*Lnpk* KO) or Cas9 protein alone (Cas9-CTL) into slices of mouse embryonic day (E)13-14 ganglionic eminences and performed live cell imaging (Extended Data Fig. 6a). We confirmed a significant reduction in *Lnpk* levels in GFP-expressing tissue microdissected from sgRNA-electroporated slices compared to controls (Extended Data Fig. 6b, *P = 0.0286). Three days after electroporation, we found that *Lnpk* KO interneurons showed a marked reduction in saltation length, saltation frequency, and speed of movement, further supporting the critical role of LNPK in interneuron migration (Extended Data Fig. 6c–f, ****P < 0.0001; ***P = 0.0002).

**Fig. 4:**
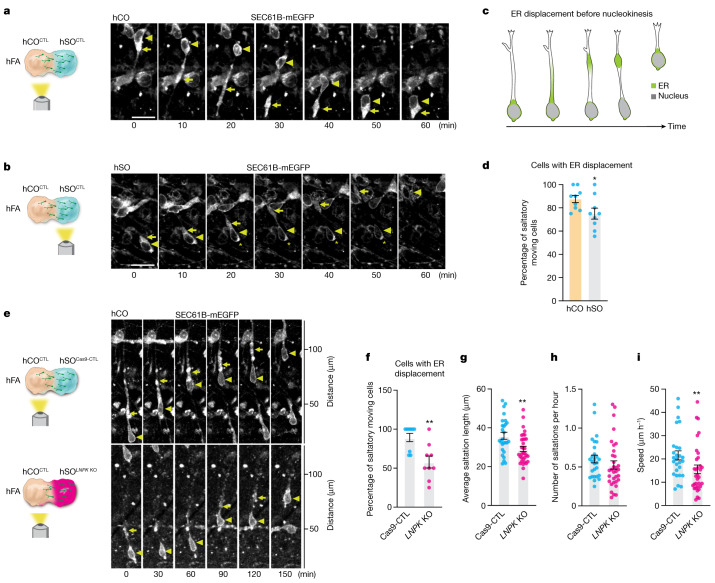
Deletion of *LNPK* impairs ER forward migration during nucleokinesis. **a**,**b**, Representative time-lapse sequences of SEC61B-mEGFP^+^ cells moving in a saltatory pattern in the hCO (**a**) and hSO (**b**) sides of the hFA (hCO is unlabelled). Images were taken at 59–80 days after hFA generation. Triangles mark the nucleus and arrows mark the linear or dilated structure of the ER in the leading branch during migration. Scale bar, 20 μm. The star in **b** indicates that this cell also shows aggregation of ER signal at the rear of the soma. **c**, A schematic depiction of ER displacement during interneuron migration. **d**, Percentages of saltatory moving cells imaged in the hCO or hSO sides of the hFA (at 59–80 days postassembly) showing ER displacement (*n* = 9 hFA for each from two differentiations). *P = 0.041, two-tailed Mann–Whitney test. **e**, Representative time-lapse sequences of SEC61B-mEGFP^+^ cells in unlabelled hCO that migrated from Cas9-CTL or *LNPK* KO hSO (in the SEC61B-mEGFP hiPS parental cell line) moving in a saltatory pattern. **f**, Percentage of SEC61B-mEGFP^+^ saltatory moving cells in the hCO side of the hFA showing ER displacement (hFA at 30–40 days after assembly. *n* = 9 hFA for each from four differentiations). **P = 0.004, two-tailed unpaired *t*-test. **g**–**i**, Saltation length (**g**), number of saltations (**h**) and speed of movement (**i**) of SEC61B-mEGFP^+^ cells in **f** (Cas9-CTL, *n* = 27 cells; *LNPK* KO, *n* = 33 cells). Two-tailed unpaired *t*-test, **P = 0.0031 (**g**). Two-tailed Mann–Whitney test, *P* = 0.1312 (**h**) and **P = 0.0079 (**i**). Data are presented as mean ± s.e.m. (**d**,**f**–**i**). Source data

**Extended Data Fig. 7:**
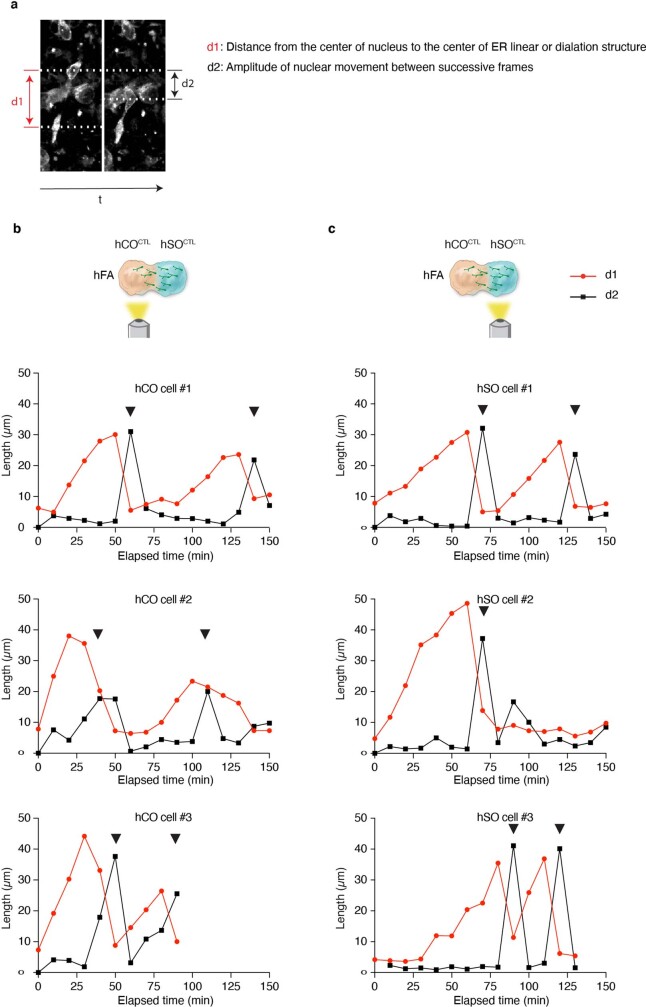
ER is displaced prior to nuclear translocation in migrating human neural cells. **a**, Example image showing measurements to study ER displacement and nuclear translocation. **b**–**c**, Relation of nuclear and ER movement in example migrating SEC61B-mEGFP^+^ cells in hFA (related to Fig. 4c). Black triangles indicate nuclear translocations.

**Extended Data Fig. 8:**
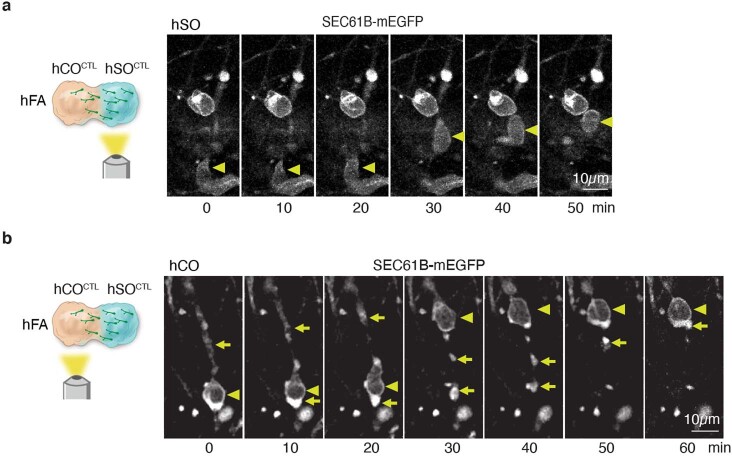
Other patterns of ER dynamics in saltatory moving cells. **a**–**b**, Time-lapse sequences of a SEC61B-mEGFP^+^ cell in hSO (**a**) or hCO (**b**) moving in a saltatory pattern. The hFA is generated by assembly of SEC61B-mEGFP hSO with unlabeled hCO. Triangles mark the nucleus and arrows mark ER localization at the leading branch or the trailing process during saltatory movement. The cell in (**a**) showed no detectable ER displacement. The cell in (**b**) has partial ER signal “left behind” the nucleus after nuclear translocation.

**Extended Data Fig. 9:**
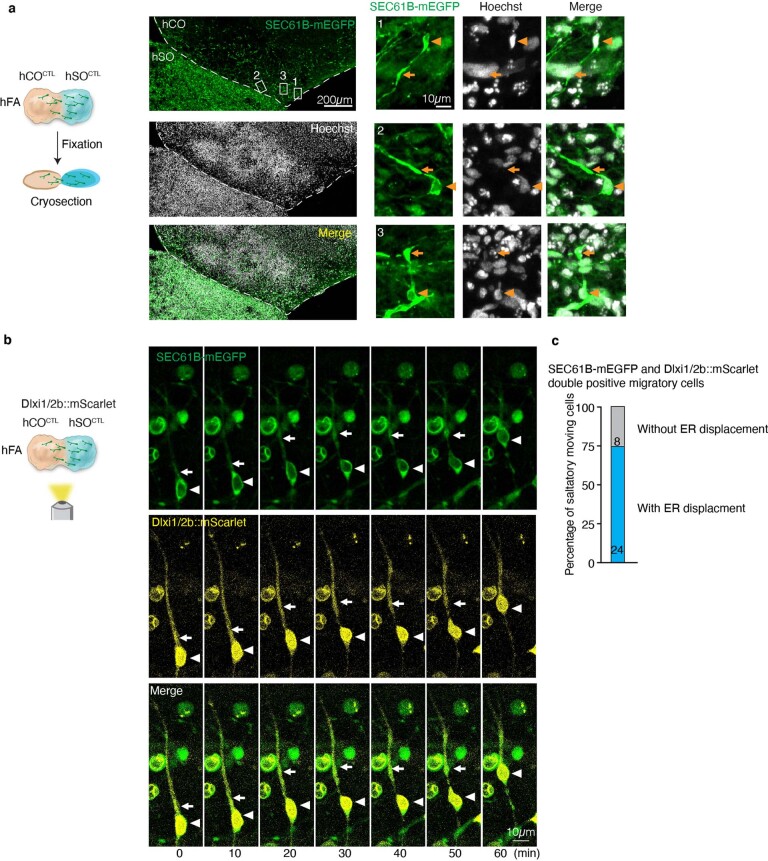
Migration-related ER displacement in fixed hFA and Dlxi1/2b::mScarlet^+^ cells. **a**, Left: Immunofluorescence cryosection images of hFA obtained by assembly of unlabeled hCO with SEC61B-mEGFP hSO. Right: Inset showing the ER distribution in SEC61B-mEGFP^+^ cells migrated into the hCO. Triangles mark the nucleus and arrows mark the linear or dilated structure of the ER in the leading branch. From 2 experiments in n = 7 hFA. **b**, Representative time-lapse sequences showing ER displacement during the saltatory movement of a SEC61B-EGFP^+^ cell that was co-labelled with Dlxi1/2b::mScarlet (at ~60 days post-assembly). Triangles mark the nucleus and arrows mark the linear or dilated structure of the ER during migration (n = 5 hFA). **c**, Percentage of SEC61B-mEGFP and Dlxi1/2b::mScarlet double positive migratory cells showing ER displacement. Live images were taken from both hCO and hSO sides (n = 5 hFA).

Whereas the role of the Golgi apparatus and the centrosome in interneuron migration has been extensively studied^39,41^ and ER changes have been observed during migration^42^, the ER has not been specifically described or directly implicated in this cellular process. This surprising ER-related gene hit (Extended Data Fig. 5l) prompted us to investigate ER dynamics in migrating human cortical interneurons. To visualize ER in migrating interneurons, we used a SEC61B-mEGFP hiPS cell line that labels the ER with mEGFP^43^. We performed confocal live imaging in hFA generated by fusing SEC61B-mEGFP hSO with unlabelled hCO to capture hSO-derived migratory cells. We found that a large fraction of the cellular ER is displaced along the leading branch in migrating cells. The ER takes on a linear conformation during this process before forming a compact structure in front of the nucleus, and this precedes nuclear translocation (Fig. 4a–c, Extended Data Fig. 7 and Supplementary Video 3). We observed this phenomenon in 75% of saltatory migrating cells in the hSO side of the hFA and in 88% of cells in the hCO side (*P = 0.041; Fig. 4d and Extended Data Fig. 8). We further confirmed our findings in fixed hFA, which showed linear or compact ER in SEC61B-mEGFP^+^ cells that migrated to the hCO side of the hFA (Extended Data Fig. 9a). To confirm that this displacement is present in migrating interneurons, we virally labelled hFA composed of SEC61B-mEGFP hSO and unlabelled hCO with Dlxi1/2b::mScarlet to label interneurons. We found that ER in Dlxi1/2b::mScarlet^+^ saltatory migrating cells showed similar displacement to the one described above (Extended Data Fig. 9b,c).

**Extended Data Fig. 10:**
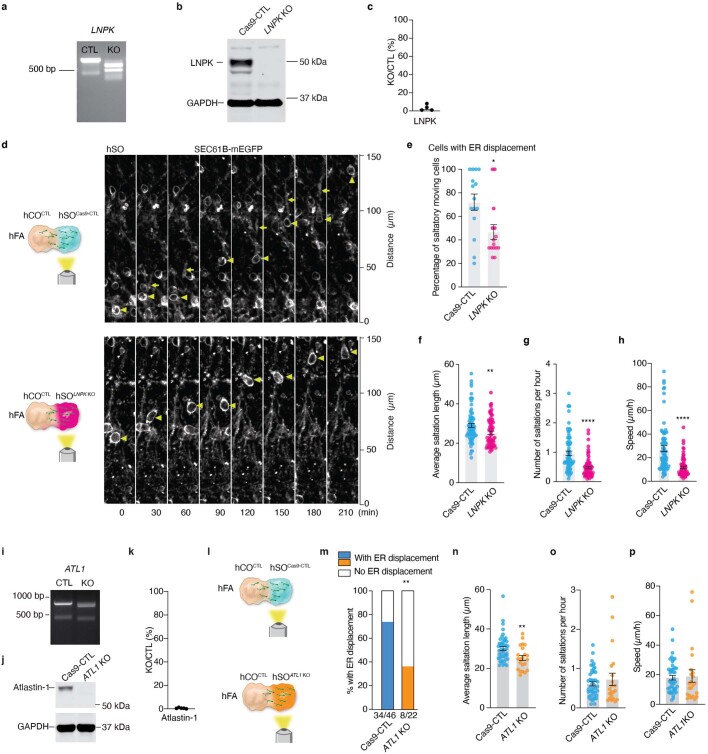
Deletion of *LNPK* or *ATL1* impaired ER displacement and saltation length of hSO saltatory migrating cells. **A**, Genotype analysis of genomic DNA extracted from hSO showing the PCR amplicon of *LNPK*. **b**, Representative western blotting image showing LNPK expression in *LNPK* KO and Cas9-CTL hSO derived from the SEC61B-mEGFP hiPS cells. GAPDH was used as a loading control. **c**, Quantification of LNPK abundance (n = 4, from 3 differentiations). **d**, Representative time-lapse sequences of SEC61B-mEGFP^+^ cells moving in a saltatory pattern in hSO assembled with an unlabeled hCO. ER displacement was detectable in the last three steps of the four in Cas9-CTL, but not in the *LNPK* KO. **e**, Percentage of SEC61B-mEGFP^+^ saltatory moving cells in the hSO side of the hFA showing ER displacement (hFA at 29–52 days after assembly. n = 15 hFA from one differentiation). * P = 0.0218, two-tailed Mann-Whitney test. **f**–**h**, Quantification of saltation length, number of saltations, and speed of movement of SEC61B-mEGFP^+^ cells in (**d**) (Cas9-CTL, n = 73 cells; *LNPK* KO, n = 68 cells). **f**, ** P = 0.0042, **g**, **h** **** P < 0.0001, two-tailed Mann-Whitney test. **i**, PCR analysis of hiPS cells showing the amplicon of *ATL1*. **j**, Representative western blotting image showing atlastin-1 abundance. GAPDH was used as a loading control. **k**, Quantification of atlatin-1 abundance (n = 5 from 4 differentiations). **l**, Schematic illustrating experimental design for performing live imaging on the hSO side of the hFA to observe saltatory migration. **m**, Percentage of saltatory migrating SEC61B-mEGFP^+^ cells showing ER displacement (Cas9-CTL, n = 46 cells from 12 hFA from 3 differentiations, 34 cells displayed ER displacement; *ATL1* KO, n = 22 cells from 9 hFA from 2 differentiations, 8 cells displayed ER displacement; two-sided Chi-square test, ** P = 0.0029). **n**–**p**, Quantification of saltation length (**n**, ** P = 0.0067), number of saltations (**o**, P = 0.5891), and speed of movement (**p**, P = 0.2646) of SEC61B-mEGFP^+^ cells in (**m**) (Cas9-CTL, n = 46 cells, from 3 differentiations; *ATL* KO, n = 22 cells from 2 differentiations); two-tailed Mann-Whitney test. Data are presented as mean ± s.e.m. (**c**, **e**, **f**–**h**, **k**, **n**–**p**). Source data

To investigate how loss of LNPK may affect ER displacement during interneuron migration, we generated *LNPK* KO hiPS cell pools (Extended Data Fig. 10a–c) with the SEC61B-mEGFP hiPS cell line and then compared ER movement of SEC61B-mEGFP^+^ migrating cells in *LNPK* KO to Cas9-CTL hSO. Investigating the migratory behaviour of hSO-derived cells in the hCO side of the hFA, we observed a lower proportion of *LNPK* KO cells showing forward ER movement before nuclear translocation compared to Cas9-CTL (Fig. 4e,f, **P = 0.004). Similar to our previous findings in the CAG::Cas9;Dlxi1/2b::eGFP hiPS cell line, we also observed reduced saltation length and slower speed of movement in SEC61B-mEGFP^+^ *LNPK* KO cells compared to Cas9-CTL (Fig. 4g–i, **P = 0.0031). To investigate whether ER displacement is also important for saltatory migration before cells move into hCO, we imaged the hSO side of the hFA and found a similar reduction in the proportion of migrating cells with ER displacement in *LNPK* KO cells compared to Cas9-CTL (Extended Data Fig. 10d,e, *P = 0.0218, and Supplementary Video 4). In addition, SEC61B-mEGFP^+^ migratory *LNPK* KO cells in hSO also showed reduced saltation length compared to Cas9-CTL (Extended Data Fig. 10f, **P = 0.0042). We also observed a reduction in the saltation frequency of *LNPK* KO cells (Extended Data Fig. 10g, ****P < 0.0001), which might be attributed to the population of cells or the side of the assembloid that was imaged (versus Fig. 4h). Collectively, this resulted in a severely impaired speed of movement in *LNPK* KO cells compared to Cas9-CTL (Extended Data Fig. 10h, ****P < 0.0001).

These data indicate that the ER migrates forward in the leading branch before nuclear translocation in migrating human cortical interneurons. Loss of LNPK results in a reduction in the proportion of cells undergoing ER displacement and may contribute to less efficient migration of *LNPK* KO interneurons in forebrain assembloids. To further verify the role of ER in interneuron migration, we deleted *ATL1*, which encodes another critical ER-shaping protein atlastin-1, in SEC61B-mEGFP hiPS cells (Extended Data Fig. 10i–k). Loss of *ATL1* in hFA resulted in a reduced proportion of saltatory cells showing ER displacement (Extended Data Fig. 10l,m, **P = 0.0029) and a reduced saltation length (Extended Data Fig. 10n–p, **P = 0.0067). Taken together, these results indicate that ER displacement before nucleokinesis is an essential step in human cortical interneuron migration.

## Discussion

Here, we have demonstrated a robust strategy to perform CRISPR loss-of-function screens for more than 400 NDD genes in hiPS cell-derived forebrain assembloids. Although a high throughput screen cannot fully reflect the complex interactions between the genetic background and the mutational effects, nor the exact gene dosage of disease-associated mutations, this platform enabled us to systematically map loss-of-function phenotypes for NDD genes onto stages of human interneuron development. Notably, it highlighted the role of cytoskeleton machinery and the ER in migration. Mutations in *LNPK*, which encodes an ER stabilizing protein^37^, cause severe intellectual disability and epilepsy in patients^38^. We found that loss of LNPK in human interneurons resulted in defects in saltatory movement. This prompted us to further investigate the dynamics of the ER during migration; we discovered a displacement of the ER in the leading branch that precedes nucleokinesis. ER-tubules interact with microtubules^44^, which are critical for establishing the leading process of migrating interneurons^45,46^. It could be that the disrupted ER-tubule structure following loss of LNPK^37^ interferes with the formation of cytoplasmic dilation in the leading neural process, which leads to a defect in cortical interneuron migration. More broadly, these experiments illustrate how mapping a list of NDD-associated genes onto cellular pathways and specific stages of human brain development could ultimately identify convergent and divergent molecular and cellular phenotypes for these conditions and facilitate therapeutic efforts^47^.

There are several limitations to our study. First, the requirement of long-term cultures and assembling organoids affects the number of genes that can be tested. Automation and strategies to accelerate interneuron maturation could facilitate the scale and the cellular features that can be screened. Second, we used hSO that resemble the MGE^6^; considering the high diversity of GABAergic interneurons, there are probably cell-specific effects of NDD genes that could not be observed using these screens. Third, there are other non-neuronal cells that actively participate in interneuron generation and migration, such as vascular endothelial cells^48^ and oligodendrocytes^49^. The current platform does not capture non-cell autonomous effects on migration mediated by loss of NDD genes in these cells. Fourth, although we determined our hits by comparing the proportional enrichment with negative control sgRNAs, we cannot exclude the possibility of having false positive or negative genes due to non-cell-autonomous effects or off-target effects of some of the sgRNAs. Last, the screens here model interneuron generation and migration towards the cortex and reflect early stages of development. Interneurons activate a program of maturation postmigration and integrate into circuits with glutamatergic neurons^50^. Future screening studies should investigate circuit assembly and final positioning of cortical interneurons.

Building on the platform described here, systematic approaches to map a large group of disease genes onto human neural development and circuit function will be essential in revealing the biology of complex NDDs and developing effective therapeutics.

## Methods

### Culture of hiPS cells

The hiPS cell lines used in this study were validated as previously described^51^. The SEC61B-mEGFP hiPS cell line (AICS-0059 cl.36) was developed at the Allen Institute for Cell Science (https://www.allencell.org/cell-catalog.html) and is distributed through Coriell^43^. The CAG::Cas9 cell line was genetically engineered in the laboratory (the parental cell line 1205-4 was derived and characterized at Stanford University^6^). The cell line was initially generated such that Cas9 expression can be turned off by adding doxycycline, but doxycycline was not used in this screen. The CAG::Cas9; Dlxi1/2b::eGFP cell line was generated in the CAG::Cas9 line. The 2242-1 control hiPS cell line used to generate hCO for the screen and the 1208-2 control hiPS cell line were derived at Stanford University. The donor for the 1205-4 line was a 30-year-old female; the donor for the 2242-1 line was a 4-year-old male; and the donor for the 1208-2 line was a 20-year-old female.

Briefly, hiPS cells were maintained in six-well plates coated with recombinant human vitronectin (Life Technologies, catalogue no. A14700) in Essential 8 medium (Life Technologies, catalogue no. A1517001). To passage hiPS cell colonies, cells were incubated with 0.5 mM EDTA for 7 min at room temperature, resuspended in Essential 8 medium and distributed into new six-well plates. Cultures were tested and maintained mycoplasma free. Approval for the derivation and use of these lines was obtained through the Stanford International Review Board. Validation of hiPS cell genome integrity was performed by high-density single-nucleotide polymorphism arrays (Supplementary Table 10).

### Generating a list of NDD genes for the screen

We compiled a list of 611 genes associated with ASD and other NDDs based on genetic studies of patients with NDD^10–13^ and the SFARI database (score 1, 2 and syndromic). To focus on NDDs genes that are expressed in hSO, we assessed gene expression in single-cell RNA sequencing data from Dlxi1/2b::eGFP^+^ cells^6^. To ensure a conservative threshold, we averaged single-cell data from each of the collected samples (from assembloids of hCO and hSO) and plotted the maximum value across samples for each measured gene. We focused on the 425 NDD genes (out of 611 total) that showed at least minimal (defined as greater than two read counts) expression in at least one averaged sample. To confirm expression in the developing human forebrain, we also analysed bulk RNA sequencing data from the ganglionic eminences (medial, caudal and lateral ganglionic eminences) of developing primary human brain at 8–9 postconception weeks using the BrainSpan. Specifically, we obtained the maximum averaged expression value (log_2_ reads per kilobase per million mapped reads, RPKM) across these samples, which is plotted in Fig. 1a. To visualize developmental expression trajectories of NDDs genes, normalized RPKM (obtained from http://development.psychencode.org/) expression values were log_2_ transformed and scaled (using the scale() function in R v.4.1.2 with default parameters). Data smoothing was performed using the geom_smooth() (as implemented in the ggplot2 package v.3.3.6) function in R with default parameters. Briefly, this function fits a penalized cubic regression spline to the data using the restricted maximum likelihood approach (by calling the mgcv:gam() R function with formula = y ~ s(x, bs = “cs”) and method = ‘REML’). To visualize the expression of screen hit genes across developing human cortex cell populations, we analysed previously published single-cell RNA sequencing data collected from mid-gestation^30^. Briefly, the raw gene count matrix was downloaded from Gene Expression Omnibus (accession number GSE162170) and processed using the standard workflow of the R package Seurat^52^ (v.4.1.0): including normalization using the sctransform function (vst.flavor = ‘v2’). Using the cell type annotations provided by the original publication, we visualized normalized gene expression of hit genes in central nervous system derived cell populations.

### Generation of the CAG::Cas9 hiPS cell line

The CAG::Cas9 line was generated as previously described^53^. The donor plasmids were constructed by modifying the *AAVS1*-CAG-hrGFP (Addgene plasmids catalogue no. 52344) and replacing the GFP with Cas9 (Addgene plasmids catalogue no. 42230), TRE and tTA (Takara, catalogue no. 63113). The plasmid containing gRNA targeting *AAVS1* locus was obtained from Addgene (plasmid no. 41818). hiPS cells (2 × 10^6^ cells) dissociated with accutase (Innovative Cell Technologies, catalogue no. AT104) were electroporated using the P3 Primary Cell 4D-NucleofectorTMX Kit L (Lonza, catalogue no. V4XP-3024), a 4D-nucleofector core unit and X unit (Lonza; nucleofection program no. CA-137). Cells were seeded on a well of a six-well plate that was precoated with vitronectin and contained prewarmed Essential 8 medium supplemented with the ROCK inhibitor Y27632 (10 µM; Selleckchem, catalogue no. S1049). Once the seeded cells reached 70–80% confluency, 1 µg ml^−1^ of puromycin in StemFlex medium (Life Technologies, catalogue no. A3349401) was supplemented for 5 days. Colonies were visible at the end of 5 days of selection in puromycin. Cells were maintained in StemFlex medium for another 5 days, and individual colonies were picked and maintained separately in a 24-well plate coated with vitronectin. Cells were maintained in StemFlex medium for another 4 days and were expanded and maintained in Essential 8 medium afterwards. Three sets of PCR programs were designed to select colonies that showed the insertion of the left, middle and right parts of the donor DNA (PCR1, 5′-TCCTGAGTCCGGACCACTTT and 3′-CACCGCATGTTAGAAGACTTCC; PCR2, 5′-TATGGAGATCCCTCGACCTG and 3′-CCTGGGATACCCCGAAGAGT; PCR3, 5′-TTCTTCTTGATGCTGTGCCG and 3′-GCTCAAGGGGCTTCATGATG). Validation of hiPS genome integrity was performed by high-density single-nucleotide polymorphism arrays.

### Generation of the CAG::Cas9;Dlxi1/2b::eGFP cell line

CAG::Cas9 hiPS cells cultured in a six-well plate were infected with lentivirus expressing Dlxi1/2b::eGFP with a hygromycin resistance gene. Cells were maintained in Essential 8 medium for another 3 days before passaging. One day after passaging, 200 µg ml^−1^ hygromycin was added to the medium to select for infected cells. Cells were maintained in hygromycin for 9 days. Control cells without virus infection died after 4 days of exposure to 200 µg ml^−1^ hygromycin. Surviving cells were expanded and cryopreserved for use.

### Generation of the sgRNA library for the screen

The interneuron migration CRISPR sgRNA library was designed by selecting up to five sgRNAs targeting each of the 438 genes of interest (425 NDD genes and 13 genes associated with interneuron development) and an extra 10% ‘safe’ harbour-targeting negative control sgRNAs (Supplementary Table 2)^54^. Cloning restriction sites and amplification primer handles were appended to each oligo. The sgRNA library oligos were synthesized on the Agilent chip oligo array synthesis platform and cloned into a lentiviral plasmid vector that constitutively expresses the sgRNA and mCherry.

### Generation of hCO and hSO from hiPS cells

hCO were differentiated from feeder-free maintained hiPS cells, as previously described^51^. To generate 3D hCO, hiPS cells were dissociated with accutase to obtain a single-cell suspension. About 3 × 10^6^ cells were seeded per AggreWell 800 plate well in Essential 8 medium supplemented with the ROCK inhibitor Y27632 (10 µM). The plate was then centrifuged at 100*g* for 3 min and incubated overnight at 37 °C with 5% CO_2_. The next day (day 0), cellular aggregates were transferred into ultra-low attachment plastic dishes (Corning, catalogue no. 3262) and cultured in Essential 6 medium (Thermo Fisher Scientific, catalogue no. A1516401) supplemented with dorsomorphin (2.5 µM, Sigma-Aldrich, catalogue no. P5499), SB-431542 (10 µM, R&D Systems, catalogue no. 1614) and XAV-939 (1.25 µM, Tocris, catalogue no. 3748). No medium change was performed on day 1. Organoids were cultured in E6 medium with patterning molecules from days 2 to 5. On day 6, organoids were transferred to neural medium containing Neurobasal A (Thermo Fisher, catalogue no. 10888022), B-27 supplement without vitamin A (Life Technologies, catalogue no. 12587), GlutaMax (1:100, Life Technologies, catalogue no. 35050061) and penicillin-streptomycin (1:100, Thermo Fisher, catalogue no. 15140163). Neural medium was supplemented with 20 ng ml^−1^ EGF (R&D Systems) and 20 ng ml^−1^ FGF2 (R&D Systems) from day 6 to 24 with daily medium change in the first 10 days, and every other day for the subsequent 9 days. From day 25 to day 43, neural medium was supplemented with 20 ng ml^−1^ brain-derived neurotrophic factor (Peprotech, catalogue no. 450-02) and 20 ng ml^−1^ NT-3 (Peprotech, catalogue no. 450-03). From day 44, only neural medium without growth factors was used for medium changes every 4 days. Gellan gum (roughly 0.005%, Sigma, catalogue no. G1919-259G, lot SLCF5726) was added in the medium from day 6 to 15 to avoid spontaneous organoid fusion^55^. The differentiation of hSO from hiPS cells is similar to hCO differentiation with the following differences in patterning: from day 6 to day 11, EGF, FGF2 and XAV-939 (1.25 µM, Tocris, catalogue no. 3748) were supplemented to the neural medium; from day 12 to day 24, EGF, FGF2, XAV-939 and SAG (100 nM, EMD Millipore, catalogue no. 566660) were used. Gellan gum (roughly 0.005%) was added in the medium from day 6 to 11 to avoid spontaneous organoid fusion.

### Generation of hFA

To generate hFA, hCO and hSO were generated separately and assembled by placing them in close proximity in 24-well low attachment plates (Corning, catalogue no. 3473), which were placed slightly tilted in the incubator^56^. Medium was changed 3 days later. Assembloids were then cultured in the 24-well low attachment plates until use, with the plates placed flat in the incubator. For the screen and the validations, day 45 hSO were fused with day 60 hCO, and hFA were used for downstream analysis at roughly 30 days postassembly. For the experiments imaging ER morphology, day 60 hSO and hCO were assembled as previously described^6^ and hFA were imaged at specific days postassembly as described.

### Interneuron generation and migration screen

CAG::Cas9;Dlxi1/2::eGFP hiPS cells were passaged with accutase 1 day before infection with the lentiviral library (with 5 µg ml^−1^ polybrene). Three days later, mCherry^+^ cells were selected by FACS (collected roughly 3.6 × 10^6^ cells) and seeded onto vitronectin coated plates to recover and expand for another 6 days. hSO were generated with these hiPS cells as described above. We observed eGFP^+^ cells at days 21–23 hSO, which is indicative of successful differentiation. We also monitored the morphology of the organoids. At day 44, hSO were collected for the interneuron generation screen. At a similar time, hFA were generated by assembling day 60 hCO generated from an unlabelled hiPS cell line with day 45 hSO. We generated a total of 1,008 assembloids. Thirty days postassembly, hFA were checked for migration under a fluorescence microscope. hFA that did not pass quality control (that is, showed low GFP signal from the hCO side) were excluded. hFA were then aligned on 100 mm plates with hSO on the left and hCO on the right, and were split at the boundary of the assembloid under a dissection scope. hCO and hSO were collected separately after checking GFP expression under a fluorescence microscope. The hSO showed strong GFP signal in the entire organoid whereas the hCO showed less GFP signal. The samples were subsequently dissociated and populations were sorted out and stored in a −80 °C freezer. We processed roughly 900 assembloids in 8 days. In addition, 5 × 10^6^ hiPS cells and day 19 organoids sampled from all plates were collected. FACS gating strategies were summarized in Supplementary Fig. 1.

### Lentivirus production

Lentivirus was produced by transfecting human embryonic kidney 293T cells with lentiviral transfer plasmids, packaging plasmids and envelope plasmids using polyethylenimine (Polysciences, catalogue no. 24765-100). Media from transfected cells was harvested at 24, 48 and 72 h after transfection. Viruses were concentrated by spinning at 17,000 rpm at 6 °C for 1 h.

### Preparation of sequencing libraries

Sample-specific sequencing libraries were prepared from the extracted genomic DNA of each collected sample by amplifying and barcoding the lentivirally integrated sgRNA spacer sequence using the Herculase II Fusion DNA Polymerase (Agilent, catalogue no. 600679). Samples were pooled and sequenced on Illumina NextSeq 550 flow cells at 450–1,000 times read depth. Bowtie v.1.2.2 was used to aligne sequencing reads.

### Analysing screen results

Screen results were analysed using the casTLE software^54^, a maximum likelihood estimator that provides for each gene: a casTLE score, a log-likelihood ratio that considers the relative enrichment or depletion of each gene-targeting sgRNA compared to the distribution of negative control sgRNA effects, a casTLE effect size, a log_2_-transformed maximum likelihood estimate of the ratio of cells containing sgRNAs perturbing a given gene in one sample compared to another, normalized to the median difference of negative control sgRNAs. In the interneuron generation screen, genes with at least two sgRNAs having effects less than or equal to −1.57 or more than or equal to +1.57, roughly twice the s.d. of negative controls and no sgRNAs with opposite effects beyond the threshold, were selected as candidates. In the interneuron migration screen, genes with at least two sgRNAs having effects less than or equal to −2.5 or more than or equal to +2.5, roughly twice the s.d. of negative controls and no sgRNAs with opposite effects beyond the threshold, were selected as candidates. To evaluate the extent of sgRNA drop out in our screen, we assessed the sequencing coverage of the sgRNAs in the final samples we collected. We found that for more than 93% of the 2,402 sgRNAs, there were at least ten sequencing counts, which can be used to calculate the enrichment of a sgRNA^54^. Furthermore, in the interneuron generation screen, more than 96% of the sgRNAs had at least 100 sequencing counts, whereas in the interneuron migration screen, more than 78% of the sgRNAs had at least 100 sequencing counts. R v.4.1.1 and Python v.3.8.5 were used to analyse sequencing data.

### Examining mutations in CAG::Cas9;Dlxi1/2b::eGFP cells transuded by lentivirus expressing sgRNAs from the screen

The sgRNAs (Supplementary Table 6) were cloned into the same lentiviral plasmid vector used in the screen that constitutively expresses sgRNA and mCherry. CAG::Cas9;Dlxi1/2b::eGFP hiPS cells were infected with lentivirus expressing each sgRNA (with 5 μg ml^−1^ polybrene). mCherry^+^ cells were enriched by FACS and sparsely seeded on a 10 cm dish followed by isolation of 48–60 clones from each infected sample. Cells (432 clones total) were cultured in 48-well plates. After one passage, genomic DNA was extracted from cells using the DNeasy 96 Blood & Tissue Kit (Qiagen, catalogue no.69581), and PCR was performed with GoTaq G2 Flexi DNA Polymerase (Promega, catalogue no. M7805) (Supplementary Table 9). PCR products were analysed by Sanger sequencing. The HET and/or homozygous state of each mutant clone was decided by ICE analysis (https://www.synthego.com/products/bioinformatics/crispr-analysis) combined with manually checking the sequence trace. Among the 432 clones we isolated, 32 clones failed to generate useful sequencing data and 31 clones were mixed clones coming from several colonies. In the 369 remining clones, there were 217 mutated clones and 152 non-mutated clones.

### Generating KO cell pools with 3× sgRNAs strategy

To validate the candidate genes of the screen, we generated KO hiPS cell pools for genes of interest with the CRISPR–Cas9 system. Three sgRNAs targeting an early exon of a specific gene were designed and synthesized by Synthego (see Supplementary Table 6 for the sgRNA sequences) to induce one or more fragment deletions (Extended Data Fig. 4b). In brief, CAG::Cas9;Dlxi1/2::eGFP hiPS cells were dissociated with accutase and 0.5 million cells were mixed with 300 pmol of sgRNAs and 40 pmol of Cas9 protein (Synthego, SpCas9 2NLS Nuclease (1,000 pmol)). Nucleofection was performed using the P3 Primary Cell 4D-NucleofectorTMX Kit L (Lonza, catalogue no. V4XP-3032), a 4D-nucleofector core unit and the X unit (Lonza, program no. CA-137). Cells were then seeded onto vitronectin coated six-well plates in Essential 8 medium supplemented with the ROCK inhibitor Y27632 (10 µM). Essential 8 medium was used for daily medium change. To generate *SMAD4* and *CSDE1* KO cells from 2242-1 and 1208-2 hiPS cells, these two control cell lines were first infected with a lentivirus expressing Dlxi1/2b::eGFP followed by hygromycin selection to stably express eGFP under the control of Dlxi1/2b. For genotyping, DNA was extracted from hiPS cells using the DNeasy Blood & Tissue Kit (Qiagen, catalogue no. 69506) and PCR was performed with GoTaq G2 Flexi DNA Polymerase (Promega, catalogue no. M7805) with primers to amplify DNA sequence around the sgRNA targeting sites of the gene of interest (Supplementary Table 9). The PCR products were separated by gel electrophoresis and Image Lab (v.6.0.1) was used for gel image acquisition.

### Generation of *SMAD4* HET hiPS cells

The *SMAD4* HET hiPS cells from the 2242-1 genetic background were generated from an individual clone isolated from the *SMAD4* KO cell pool. To generate *SMAD4* HET lines in the 1205-4 genetic background, CAG::Cas9;Dlxi1/2::eGFP hiPS cells were dissociated with accutase and 0.5 million cells were mixed with 40 pmol of sgRNA (AAAAAGAGCAAUUGAAAGUU) and 5 µg of Cas9 protein (Integrated DNA Technologies (IDT), Alt-R S.p. Cas9 Nuclease V3). Nucleofection was performed in the same way as described above. Individual clones were isolated, and the HET line was confirmed by Sanger sequencing.

### Preparing samples for next-generation sequencing

DNA was isolated from hSO using the DNeasy Blood & Tissue Kit (Qiagen, catalogue no. 69506). Primers (Supplementary Table 7) with Illumina adaptors were used to amplify roughly 300 bp of DNA sequence around the sgRNA targeting site of the gene of interest. PCR was performed with the PrimeSTAR Max DNA polymerase 2× hot-start PCR master mix (TaKaRa Bio, catalogue no. R045A). PCR products were purified using the AMPure XP PCR purification system (Beckman Coulter, catalogue no. A63881) and the 96S Super Magnet (Alpaqua, catalogue no. A001322) for next-generation sequencing (Azenta).

### Dissociation of hCO and hSO

hCO and hSO were dissociated as previously described^57^ with some modifications. Briefly, organoids (single organoids cultured in a 96-well plate or up to eight organoids cultured in a six-well plate) were incubated with an enzyme solution containing 10 U ml^−1^ papain (Worthington, catalogue no. LS003127), 1× Earle’s balanced salt solution (Sigma-Aldrich, catalogue no. E7510-500ML), 0.36% d(+)-glucose (Sigma-Aldrich), 26 mM NaHCO_3_ (Sigma-Aldrich), 0.5 mM EDTA (Sigma-Aldrich), DNase (Worthington, catalogue no. LS002007), 6.1 mM l-cysteine (Sigma-Aldrich) and the ROCK inhibitor Y27632 (10 µM) at 37 °C with 5% CO_2_ for 15 min; this solution was prewarmed at 37 °C for 30 min to activate papain, and then cells were washed with a prewarmed protease inhibitor solution (1× Earle′s balanced salt solution, 0.36% d(+)-glucose, 26 mM NaHCO_3_, 0.2% trypsin inhibitor (Worthington, catalogue no. LS003086)). Organoids were then triturated, and the resulting single-cell suspension was centrifuged at 1,200 rpm for 5 min. The pellet was resuspended with 3% BSA (Sigma-Aldrich) and then filtered.

### Flow cytometry

To sort mCherry^+^ or GFP^+^ cells during the screen, FACS was conducted on BD-Aria II instruments (Stanford FACS Facility). The ACEA NovoCyte Quanteon 4025 flow cytometer (Stanford FACS Facility) was used to analyse the percentage of GFP^+^ cells. NovoExpress (v.1.3.0) was used to generate flow cytometry data. Data were analysed on FlowJo (v.10.8.1).

### Real-time quantitative PCR

For real-time qPCR analysis, 2–3 organoids were pooled as a sample. mRNA was isolated using the RNeasy Mini Plus kit (Qiagen, catalogue no. 74136) and template complimentary DNA was prepared by reverse transcription using the SuperScript III First-Strand Synthesis SuperMix for RT–qPCR (Thermo Fisher Scientific, catalogue no. 11752250). Real-time qPCR was performed using the SYBR Green PCR Master Mix (Thermo Fisher Scientific, catalogue no. 4312704) on a QuantStudio 6 Flex Real-Time PCR System (Thermo Fisher Scientific, catalogue no. 4485689). Data were processed using the QuantStudio RT–PCR software (Applied Biosystems, v.1.7.1). To plot the heatmap to present the qPCR results, gene expression was scaled and mean centred (using the ‘scale’ function in R) and the heatmap was plotted using the ‘ComplexHeatmap’ package. Primers used in this study are listed in Supplementary Table 8.

### Clearing, staining and imaging of hFA

The Clear, Unobstructed Brain/Body Imaging Cocktails and Computational analysis (CUBIC) method was applied to optically clear hFA, as previously described^58^. Briefly, hFA at roughly 40 days postassembly were fixed overnight with 4% paraformaldehyde (PFA) at 4 °C. The next day, samples were washed with PBS (2 h × 3, 37 °C) before incubation with the Tissue-Clearing Reagent CUBIC-L (TCI, catalogue no. T3740) at 37 °C for roughly 18 h. After washing with PBS (2 h × 3, 37 °C), samples were incubated in a nuclear staining solution (1:150 dilution of RedDot, Biotium 40061-T) in PBS with 500 mM NaCl at 37 °C overnight. Samples were then washed with PBS (2 h × 2, 37 °C) followed by incubation with HEPES-TSB (10 mM HEPES, 10% TX-100, 200 mM NaCl, 0.5% BSA) buffer at 37 °C for 2 h. Samples were subsequently stained with anti-GFP antibody (GTX13970, 1:1,000) for 48 h and secondary antibody (Alexa Fluor 488 AffiniPure Donkey Anti-Chicken IgY, Jackson ImmunoResearch catalogue no. 703-545-155, 1:300) for another 48 h. Antibodies were prepared in HEPES-TSB buffer and hFA were washed with 10% TX-100 in PBS (2 h × 2, 37 °C) and HEPES-TSB (2 h × 1, 37 °C) in between stains. After secondary antibody staining, samples were washed with 10% TX-100 in PBS (30 min × 2, 37 °C) and PBS (1 h × 1, 37 °C) followed by subsequent incubation with 50 and 100% Tissue-Clearing Reagent CUBIC-R+ (TCI, catalogue no. T3741) at room temperature for 1 day each. All incubation steps were performed on a shaker. Samples were transferred to a 96-well plate with a clear bottom (ibidi, catalogue no. 89626) in CUBIC-R+ solution and imaged using a ×10 objective on a Leica Stellaris confocal microscope. The entire hCO and the hCO-hSO boundary were imaged (roughly 1 mm volume per hFA) blindly to the genotype of the samples.

Images were analysed with Imaris (Oxford Instruments, v.9.7.0) and Fiji (ImageJ, v.1.0 and v.1.53f51). Briefly, Z-stack confocal images were compiled in Imaris to generate a 3D reconstruction of the hFA. The boundary of the assembloid in individual frames was manually delineated using the nuclear stain signal (roughly every 40 frames); these boundaries were used to create an Imaris ‘surface’ to define the hCO (Supplementary Video 1). The 3D hCO was then reduced into a two-dimensional (2D) projection using the maximum intensity function in Fiji. To measure the Dlxi1/2b::eGFP intensity in a consistent manner for all samples, the hCO-hSO boundary was aligned vertically. One third of the hCO adjacent to the boundary was used to quantify mean intensity. A representative area of background noise was also measured for mean intensity and was subtracted from the measurements of the hCO.

### Western blotting

Whole cell protein lysates were prepared using the RIPA buffer system (Santa Cruz, catalogue no. sc-24948) for LNPK, SYNCRIP and TERF2 blots. For CSDE1 and SMAD4, whole cell protein lysates from samples were prepared using SDS Buffer (1.5% SDS, 25 mM Tris pH 7.5). Briefly, 50 µl of SDS buffer was added to two spheres in a 1.5 ml tube. Samples were sonicated for a total of 7–9 s using an ultrasonicator (Qsonica Q500 sonicator; pulse 3 s on, 3 s off; amplitude 20%) using a small tip attachment until samples were no longer viscous. Protein concentration was quantified using the bicinchoninic acid assay (Pierce, Thermo Fisher, catalogue no. 23225). For electrophoresis, 20 μg of protein per lane was loaded and run on a 4–12% Bis-Tris PAGE gel (Bolt 4–12% Bis-Tris Protein Gel, Invitrogen, catalogue no. NW04122BOX) and transferred onto a polyvinyl difluoride membrane (Immobilon-FL, EMD Millipore). Membranes were blocked with 5% BSA in tris-buffered saline with Tween (TBST) for 1 h at room temperature and incubated with primary antibodies against TERF2 (rabbit 1:2,000, Novus Biologicals, catalogue no. NB110-57130SS), SYNCRIP (mouse 1:2,000, MilliporeSigma, catalogue no. 05-1517), Atlastin-1 (mouse, 1:500, Sigma, catalogue no. MABN1831, Clone 3194) and GAPDH (glyceraldehyde 3-phosphate dehydrogenase) (mouse, 1:5,000, GeneTex, catalogue no. GTX627408) overnight and LNPK (rabbit, 1:500, Sigma, catalogue no. HPA014205), CSDE-1 (rabbit, 1:500, Abcam, catalogue no. ab201688), and SMAD4 (mouse 1:250, Santa Cruz Biotechnology, catalogue no. sc-7966) for 72 h at 4 °C. Membranes were washed three times with TBST and then incubated with near-infrared fluorophore-conjugated species-specific secondary antibodies: Goat Anti-Mouse IgG Polyclonal Antibody (IRDye 680RD, 1:10,000, LI-COR Biosciences, catalogue no. 926–68070) or Goat Anti-Rabbit IgG Polyclonal Antibody (IRDye 800CW, 1:10,000, LI-COR Biosciences, catalogue no. 926–32211) for 1 h at room temperature. Following secondary antibody application, membranes were washed three times with TBST, once with TBS and then imaged using a LI-COR Odyssey CLx imaging system (LI-COR) with Image studio software (Licor, v.5.2). Western blots were analysed using Image studio software (Licor, v.5.2) or ImageJ (v2.3.0). See Source Data for full blots.

### Immunofluorescence staining

hiPS cells were fixed in 4% PFA-PBS for 20 min at room temperature. hSO and hFA were fixed in 4% PFA-PBS overnight at 4 °C followed by dehydration in 30% sucrose for 3 days. Subsequently, samples were embedded in optimal cutting temperature (OCT) compound (Tissue-Tek OCT Compound 4583, Sakura Finetek) and 30% sucrose–PBS (1:1) for cryosectioning (25-μm-thick sections) using a Leica Cryostat (Leica, catalogue no. CM1860). For immunofluorescence staining, cryosections were washed with PBS to remove excess OCT. Fixed hiPS cells and cryosections were blocked in 10% normal donkey serum (NDS) (MilliporeSigma, catalogue no. S30-M) and 0.3% Triton X-100 (MilliporeSigma, catalogue no. T9284-100ML) diluted in PBS for 1 h at room temperature. Sections were then incubated overnight at 4 °C with primary antibodies diluted in PBS containing 10% NDS and 0.3% Triton X-100. PBS was used to wash away excess primary antibodies, and the cryosections were incubated with secondary antibodies in PBS containing 10% NDS and 0.3% Triton X-100 for 1 h. The following primary antibodies were used for staining: anti-Cleaved Caspase-3 (Asp175) antibody (1:500 dilution, rabbit, Cell Signaling Technology, catalogue no. 9661), anti-SOX2 antibody (1:200 dilution, goat, R&D, catalogue no. AF2018), anti-NeuN antibody (1:200 dilution, mouse, Abcam, catalogue no. ab104224), anti-GFP antibody (1:1,000 dilution, chicken, GeneTex, catalogue no. GTX13970) and anti-LNPK antibody (1:500, rabbit, Sigma, catalogue no. HPA014205). Alexa Fluor 488 AffiniPure Donkey Anti-Chicken IgY (Jackson ImmunoResearch, catalogue no. 703-545-155) and Alexa Fluor 568 donkey anti-rabbit IgG (H&L) highly cross-adsorbed secondary antibody (Thermo Fisher Scientific, catalogue no. A10042), Alexa Fluor Plus 647 donkey anti-goat IgG (H&L) highly cross-adsorbed secondary antibody (Thermo Fisher Scientific, catalogue no. A32849) and Alexa Fluor 647 donkey anti-mouse IgG (H&L) highly cross-adsorbed secondary antibody (Thermo Fisher Scientific, catalogue no. A31571) were used in 1:1,000 dilution. The nuclei were visualized with Hoechst33258 (Thermo Fisher Science, catalogue no. H3569, 1:10,000 dilution). Fixed hiPS cells and cryosections were mounted for microscopy on glass slides using Aquamount (Polysciences, catalogue no. 18606) and imaged on a Leica Stellaris confocal microscope. LAS-X (Leica) software was used for acquiring the microscopy data. Images were processed with Fiji (ImageJ, v.1.0, v.2.3.0). The fraction of cCasp3^+^ hiPS cells was quantified by imaging three randomly selected colonies from each independent experiment. The fraction of cCasp3^+^, SOX2^+^ and NeuN^+^ cells in hSOs was quantified by imaging at least three sections from each individual hSO. Following background subtraction, nuclei were identified using a threshold and watershed algorithm on the Hoechst channel. The mean fluorescence intensity within identified nucleus regions was calculated for each channel. The same cutoff was used for samples from the same batch to distinguish positive cells. The data from many sections of the same organoid were averaged and then presented in the figure.

### Live cell imaging and analysis of Dlxi1/2b::eGFP^+^ cell migration

To image the Dlxi1/2b::eGFP^+^ cell migration, hFA were transferred to a well of a 96-well plate with a clear bottom (ibidi, catalogue no. 89626) in 500 µl of neural medium under environmentally controlled conditions (37 °C, 5% CO_2_). Samples were placed in this condition for 30–60 min before imaging on a confocal microscope (Leica, catalogue no. SP8). hFA were imaged under a ×10 objective at a depth of 50–150 μm and at a rate of roughly 20 min per frame for 18–22 h. Fiji (ImageJ, v.1.0) was used to quantify the migration of Dlxi1/2b::eGFP^+^ cells. To estimate the length of individual saltations, Dlxi1/2b::eGFP^+^ cells showing a swelling of the soma were identified, and the distance (in μm) to the new position of the soma following nucleokinesis was measured manually. The time necessary for this movement was used to calculate saltation frequency. Only cells that showed at least two complete jumps during the imaging session were included in the analysis, and the averaged saltation length of each cell was used in the statistical analysis. Analysis was carried out blindly to the genotype.

### Live cell imaging and analysis of SEC61B-mEGFP^+^ cell migration

To image the SEC61B-mEGFP^+^ cells, hFA (SEC61B-mEGFP^+^ CTL, CAS9-CTL, *LNPK* KO, or *ATL1* KO hSO assembled with unlabelled hCO) were imaged under environmentally controlled conditions (37 °C, 5% CO_2_) on a confocal microscope (Leica Stellaris, ×20 objective, ×2 zoom). The samples were placed in the controlled environment for 1 h before imaging. Samples were imaged at a depth of roughly 12 μm and a rate of 10 min per frame for roughly 20 h. Given the short working distance of the ×20 objective and the nature of 3D floating samples, only images at the very bottom of the samples were used. One to three images per hFA were obtained for the analysis. Images that contain densely packed fast-moving cells were excluded. To analyse the percentage of saltatory migrating cells in hCO or hSO showing ER displacement, SEC61B-mEGFP^+^ cells showing saltatory migrations with at least two jumps in the imaging session were selected and cells showing a swelling of the ER at the leading neural branch preceding nuclear translocation were identified as showing ER displacement (cells were considered to have this ER displacement if one of the jumps showed this displacement). The hCO or hSO with at least three saltatory moving cells were included to analyse ‘the percentage of cells with ER displacement’. When estimating the saltation length and saltation frequency of SEC61B-mEGFP^+^ migratory cells, a strategy similar to that used for measuring Dlxi1/2b::eGFP^+^ migrating cells was used. Cells with at least two complete jumps were included. Fiji (ImageJ, v1.0 and v1.53f51, and ImageJ2, v.2.3.0/1.53q) was used to analyse the migration of SEC61B-EGFP^+^ cells. Blinding for genotype was used for analysis.

When examining Dlxi1/2b::mScarlet virally labelled hFA (SEC61B-mEGFP hSO assembled with unlabelled hCO), Dlxi1/2b::mScarlet^+^ cells were identified by the florescence signal filling the entire soma and extending to the leading branch (SEC61B-mEGFP hiPS line also expresses mTagRFP-T tagged LMNB1, which was captured while imaging mScarlet). ER displacement was examined on the basis of the SEC61B-mEGFP signal as described above.

### Viral labelling for live imaging

hFA (SEC61B-mEGFP hSO assembled with unlabelled hCO) were infected with lentivirus encoding Dlxi1/2::mScarlet. Briefly, 30 days postassembly, hFA cultured in 24-well low attachment plates with 500 μl of neural medium were cultured with virus overnight at 37 °C with 5% CO_2_. The next day, fresh neural medium (500 μl) was added. The following day, the medium was replaced with fresh medium. Samples were ready for live imaging 2 weeks after infection.

### ASO

ASO used in this study was manufactured by IDT (/52MOErC/*/i2MOErT/*/i2MOErT/*/i2MOErC/*/i2MOErC/*A*A*C*C*C*A*T*A*A*T*/i2MOErT/*/i2MOErT/*/i2MOErT/*/i2MOErT/*/32MOErG/). ASO was reconstituted in nuclease-free water at a concentration of 1 mM and stored at −20 °C thereafter. In the experiment, 5 μM ASO diluted with neural medium was applied to hFA.

### Mouse slice electroporation

Brains from E13-E14 CD1 mouse embryos (not genotyped for sex) were dissected, embedded in 4% low melting temperature agarose and subsequently sectioned in Hanks’ balanced salt solution (HBSS) supplemented with glucose (100 ml of 10× HBSS without Ca or Mg, 2.5 ml of 1 M HEPES buffer at pH 7.4, 30 ml of 1 M d-glucose, 10 ml of 100 mM CaCl_2_, 10 ml of 100 mM MgSO_4_ and 4 ml of 1 M NaHCO_3_) or ACSF (125 mM NaCl, 2.5 mM KCl, 1 mM MgCl_2_, 2 mM CaCl_2_, 1.25 mM NaH_2_PO_4_, 25 mM NaHCO_3_, 25 mM glucose) bubbled with 95% oxygen and 5% carbon dioxide using a Leica vibratome. Next, 300 μm coronal slices with readily discernible forebrain structures were placed into tissue culture dishes containing slice culture media (31% HBSS, 60% basal media Eagle, 5% FBS, 1% glucose, 1% N2 Supplement (Thermo Fisher Scientific), 1% GlutaMax (Thermo Fisher Scientific) and 1% penicillin-streptomycin) for electroporation. Using a sterile, pulled and bevelled glass micropipette, ganglionic eminences were locally coinjected with a CAG-driven GFP plasmid (pCAG-IRES-GFP, gift from C. Cepko through Addgene, plasmid no. 11159, ref. ^59^) mixed with either a sgRNA–Cas9 complex (Synthego) directed to mouse *Lnpk* or Cas9 protein alone. Randomly picked slices were electroporated with horizontally oriented paddle electrodes at 50 V using a BTX Square Pulse electroporator and were subsequently grown at the air liquid interface on Millicell cell culture inserts (Millipore catalogue no. PICM03050) for 3 days until live imaging or real-time qPCR. To determine the efficiency of *Lnpk* knockdown, slices were carefully microdissected to isolate regions containing EGFP-expressing cells, which were then placed directly in lysis buffer for RNA extraction (Qiagen RNeasy Micro Kit). Animal experiments were performed in accordance with National Institutes of Health (NIH) guidelines for the care and use of laboratory animals under protocols approved by the Stanford University’s Administrative Panel on Laboratory Animal Care and University of California, San Francisco Institutional Animal Care and Use Committees. Mice were housed in an animal facility on a 12 h/12 h light/dark cycle, with 20–22 °C (68–72 °F) and 30–70% humidity. See Source Data 5 for the data used to generate bar graphs in Extended Data Fig. 6.

### Statistics

Data are analysed with GraphPad Prism (v.9.1.0) unless otherwise indicated. Data are presented as mean ± s.e.m., unless otherwise indicated. Distribution of the raw data was tested for normality of distribution; statistical analyses were performed using the Student’s *t*-test, Mann–Whitney test, Kruskal–Wallis test and one- or two-way analysis of variance (ANOVA) with multiple comparison tests as indicated. Sample sizes were estimated empirically. Blinding of genotypes was used for imaging analyses, flow cytometry analysis and organoid size measurements.

### Reporting summary

Further information on research design is available in the Nature Portfolio Reporting Summary linked to this article.

## Online content

Any methods, additional references, Nature Portfolio reporting summaries, source data, extended data, supplementary information, acknowledgements, peer review information; details of author contributions and competing interests; and statements of data and code availability are available at 10.1038/s41586-023-06564-w.

## Supplementary information

Supplementary Information Supplementary Fig. 1 and Tables 1–10.

Reporting Summary

Supplementary Data Source Data 1–4.

Supplementary Video 1 *Z*-stack images of cleared hFA and image processing to examine interneuron migration.

Supplementary Video 2 Live imaging showing migration of Dlxi1/2b::eGFP^+^ cells in fused hFA derived from Cas9-CTL or LNPK KO hSO fused with unlabelled hCO.

Supplementary Video 3 Live imaging showing a saltatory migrating SEC61B-mEGFP^+^ cell in fused hFA with ER forward migration in the leading branch before nuclear translocation.

Supplementary Video 4 Live imaging showing migration of SEC61B-mEGFP^+^ cell in fused hFA derived from Cas9-CTL or LNPK KO hSO fused with unlabelled hCO.

## Source data

Source Data Fig. 2

Source Data Fig. 3

Source Data Fig. 4

Source Data Extended Data Fig. 1

Source Data Extended Data Fig. 4

Source Data Extended Data Fig. 5

Source Data Extended Data Fig. 6

Source Data Extended Data Fig. 10

## Data Availability

The data that support the findings of this study are available on request from the corresponding author. The following public dataset were used to support this study: the SFARI database (https://gene.sfari.org). The single-cell RNA sequencing data from Dlxi1/2b::eGFP^+^ cells were generated by Birey et al.^6^ and were downloaded from Gene Expression Omnibus under accession number GSE93811. Trevino et al.^30^ data were downloaded from the Gene Expression Omnibus with the accession number GSE162170. Bulk RNA sequencing data from the developing human cortex were generated by psychENCODE (BrainSpan, downloaded from http://development.psychencode.org/). Source data are provided with this paper.

## Code availability

The codes for casTLE analysis can be downloaded at https://bitbucket.org/dmorgens/castle/downloads/. For the analysis of publicly available RNA sequencing data, no custom software algorithms were generated for this study. All analyses were carried out using standard software packages with default parameters.
