# Supplementary material for: Assembloid CRISPR screens reveal impact of disease genes in human neurodevelopment
Source: Nature. 2023 Sep 27;622(7982):359–66. doi: 10.1038/s41586-023-06564-w (PMC10567561; doi:10.1038/s41586-023-06564-w)
Supplement: Supplementary file 1 — Supplementary Fig. 1 and Tables 1–10. [file 41586_2023_6564_MOESM1_ESM.pdf]

nature portfolio
https://doi.org/10.1038/s41586-023-06564-w

Supplementary information

Assembloid CRISPR screens reveal impact of disease genes in human neurodevelopment

In the format provided by the authors and unedited

Supplementary Table 1. List of 611 NDDs genes

ARX	*GRIN2B*	*RLIM*	*SYNGAP1*
CREBBP	*ICA1*	*DHX30*	*PHF2*
KMT2E	*JARID2*	*CDK8*	*SHANK3*
PPP5C	*CSDE1*	*SNX14*	*MAGEL2*
SPAST	*ZMYND11*	*AHI1*	*PON1*
NRXN3	*CUL7*	*RAC1*	*UBE3C*
CUL3	*SPEN*	*GABBR2*	*NFE2L3*
TRIO	*PHRF1*	*FBXO11*	*DLX3*
AP2S1	*P4HA2*	*GALNT2*	*ASPM*
ARID1B	*CLASP1*	*LNPK*	*DAPP1*
NEXMIF	*KCNQ2*	*MED12L*	*NUAK1*
KMT2C	*CTTNBP2*	*HDAC8*	*GPC4*
NCKAP1	*OPHN1*	*TAF1*	*RBFOX1*
RFX3	*KAT6A*	*NFIB*	*P2RX5*
SRCAP	*ACHE*	*NTRK2*	*LAMB1*
NCOA1	*SNX5*	*POLR3A*	*MYO9B*
ATRX	*RBM27*	*PAK1*	*PRODH*
POMGNT1	*SUPT16H*	*CHD1*	*INTS6*
CNOT3	*AGO1*	*XPC*	*SLC7A5*
DPYSL2	*BTAF1*	*SLC45A1*	*PRKD2*
WAC	*SBF1*	*WDR26*	*GRIK5*
LZTR1	*TNRC6B*	*ATP1A1*	*MET*
EP300	*NINL*	*RAD21*	*CNTNAP3*
CHD8	*RAB2A*	*KIF5C*	*DOCK8*
ZMYND8	*UBR5*	*YWHAG*	*KAT2B*
ADNP	*ETFB*	*PPM1D*	*PARD3B*
TM9SF4	*TAF6*	*GATM*	*AMPD1*
CELF4	*CEP41*	*HCFC1*	*KIF14*
FMR1	*KDM4C*	*RSRC1*	*USP45*
CORO1A	*ZMIZ1*	*PCCA*	*PREX1*
CTCF	*ELP4*	*DOLK*	*VIL1*
TSC2	*ERBIN*	*NR2F1*	*WDFY4*
BCKDK	*SEMA5A*	*RIMS2*	*CGNL1*
MED13	*CCNG1*	*RPS6KA3*	*GALNT8*
RAI1	*CPEB4*	*PACS2*	*CASZ1*
PHF12	*CCT4*	*SGSH*	*EFR3A*

KMT5B	*SRSF11*	*AP1S2*	*CNTN6*
NRXN2	*APH1A*	*TRAPPC6B*	*TRPM1*
ALDH5A1	*CNR1*	*PRKD1*	*ANXA1*
PPP2R5D	*SET*	*ANKS1B*	*AGAP2*
NUP155	*SLC35B1*	*TET3*	*CIB2*
TRIM23	*LRP1*	*ARID2*	*LMX1B*
UBE3A	*SLC12A5*	*TRRAP*	*TRPC6*
FOXP1	*RPS10P2-AS1*	*CEP290*	*ADCY3*
HDLBP	*KDM5C*	*POU3F3*	*HECW2*
SON	*HIVEP3*	*DMD*	*PRICKLE1*
ASH1L	*SMARCA4*	*VAMP2*	*SLC6A3*
KDM5B	*AKAP9*	*KCNMA1*	*KIRREL3*
KMT2A	*ZC3H4*	*MSL3*	*TAOK2*
PHF3	*CHMP1A*	*PDK2*	*CNTN5*
IRF2BPL	*CC2D1A*	*TNPO3*	*CNTNAP4*
DNMT3A	*PER2*	*SMARCD1*	*FAM92B*
BCL11A	*TERF2*	*GNAO1*	*GRIP1*
KANSL1	*MYH10*	*CABP7*	*ATP2B2*
GRIA2	*UNC79*	*CSNK2A1*	*SHANK1*
KDM3B	*AGO4*	*NAA10*	*DISC1*
MED13L	*CAPRIN1*	*MAPK3*	*PYHIN1*
BAZ2B	*USP15*	*RAB11A*	*STXBP5*
UPF3B	*DENR*	*PPP2R1A*	*SLITRK5*
MBOAT7	*ST8SIA2*	*TNPO2*	*FBN1*
HNRNPH2	*CDH13*	*DNM1*	*AVPR1A*
GNAI1	*NCOR1*	*LARP4B*	*CHRNA7*
EIF3G	*SMAD4*	*ENO3*	*OTUD7A*
ARHGEF9	*SAE1*	*EFTUD2*	*ZNF804A*
TRAF7	*ILF2*	*NSD2*	*KCNS3*
KDM6B	*CNTN4*	*CHD4*	*PAH*
VPS13B	*AMT*	*CERT1*	*RAB43*
MEIS2	*NLGN4X*	*HMGXB3*	*CEP135*
SOX5	*KDM6A*	*PAPOLG*	*GRIN1*
PHF21A	*ASTN2*	*SF3B1*	*KCNJ10*
VEZF1	*CNKSR2*	*ODC1*	*OXTR*
SCN2A	*DLG2*	*AGO3*	*GABRG3*
STXBP1	*DIP2C*	*ZC4H2*	*GRID1*
SMARCC2	*ASAP2*	*KIF1A*	*GRIN2A*
ASXL3	*SPARCL1*	*PDHA1*	*PRKN*

RERE	*GRIA1*	*RRP8*	*SHOX*
POGZ	*TBC1D31*	*FAM104A*	*TMLHE*
SCN1A	*CACNA1D*	*SYNCRIP*	*WWOX*
TBCK	*SLC38A10*	*TRA2B*	*CACNA1H*
ANK2	*UBN2*	*ABI2*	*ADA*
GABRB2	*EMSY*	*HNRNPD*	*OR52M1*
PHIP	*PRICKLE2*	*TCF12*	*ATP10A*
GIGYF1	*PLXNB1*	*ANP32A*	*PTCHD1-AS*
TLK2	*GRIK2*	*CLTC*	*MFRP*
CASK	*SLC7A3*	*MTF2*	*MSNP1AS*
ZNF462	*CACNB2*	*SUSD4*	*ITGB3*
LRRC4C	*APBB1*	*GATAD2B*	*CYFIP1*
CACNA1C	*LEO1*	*UNC80*	*MIR137*
ANK3	*PRKCB*	*BTF3*	*GGNBP2*
SETBP1	*NAV2*	*NONO*	*NFIX*
HNRNPU	*PHB*	*TMEM178A*	*CDK13*
NR4A2	*TRAPPC9*	*PRKCA*	*HDAC4*
TRIP12	*KIAA1586*	*KAT6B*	*BCORL1*
SLC6A1	*TET2*	*KCNJ6*	*SETD1A*
CACNA2D3	*SCN9A*	*STC1*	*DEPDC5*
DYRK1A	*NLGN1*	*STARD9*	*CHKB*
BRAF	*CTNND2*	*DVL3*	*EEF1A2*
AUTS2	*RALGAPB*	*RPL26*	*PIK3R2*
UBR1	*DLGAP1*	*PRPF18*	*HOXA1*
DIP2A	*NUDCD2*	*SRRM2*	*CUX2*
TAOK1	*GPHN*	*H1-4*	*FBRSL1*
SHANK2	*MACROD2*	*MAP2K1*	*KPTN*
WDFY3	*CNTNAP2*	*TMEM42*	*SATB2*
NAA15	*DPP10*	*KCND3*	*ZSWIM6*
NIPBL	*PAK2*	*ASXL1*	*CYP27A1*
PTCHD1	*PLCB1*	*SNAPC5*	*SETD1B*
NSD1	*EP400*	*PUF60*	*CACNA1A*
TSC1	*ROBO2*	*SKIDA1*	*SIK1*
GABRB3	*USP7*	*PSMG4*	*TTN*
MAP1A	*MYO5A*	*PBX1*	*NTNG1*
ANKRD11	*QRICH1*	*YTHDF3*	*HCN1*
CTNNB1	*CDC42BPB*	*SEPTIN10*	*INTS1*
SETD5	*MTOR*	*WDR45*	*BRWD3*
MECP2	*RANBP17*	*PPP1CB*	*HEPACAM*

SIN3A	*HMGN1*	*FAM200A*	*TM4SF20*
NLGN2	*PLXNA4*	*FAM200B*	*FRMPD4*
CHD3	*CUX1*	*ALG13*	*PHF8*
KIAA0232	*RALA*	*SNAP25*	*RNF135*
TANC2	*PAX6*	*SLC35A2*	*TBX1*
CHD7	*GABRA3*	*CDKL5*	*ALDH1A3*
PTEN	*TBC1D23*	*HIVEP2*	*NTNG2*
DHCR7	*CAMK2B*	*CIC*	*DLL1*
NBEA	*CTNNA2*	*RIMS1*	*ACY1*
HECTD4	*SYT1*	*TCF20*	*TAB2*
CHD2	*RORA*	*DMPK*	*U2AF2*
HRAS	*CAMK2A*	*EBF3*	*FOSL2*
PACS1	*SMC1A*	*PPP1R9B*	*GLRA2*
FOXG1	*CLCN4*	*PTK7*	*CAPN15*
DEAF1	*ACTB*	*TEK*	*ADAP1*
TBL1XR1	*ACTL6B*	*TSHZ3*	*CBL*
PTPN11	*SMARCA2*	*IQSEC2*	*C2orf42*
NRXN1	*MEF2C*	*PRR12*	*PIK3CA*
EHMT1	*HUWE1*	*AHDC1*	*KCNC1*
SETD2	*ALG6*	*FOXP2*	*SPRY2*
ZBTB20	*CCNK*	*GFAP*	*KIF11*
SRPRA	*YY1*	*DLG4*	*KCNH1*
MYT1L	*NOVA2*	*TBR1*	*AQP10*
ZNF292	*ATP1A3*	*TCF7L2*	*ITPR1*
NLGN3	*RHEB*	*MKX*	*BRPF1*
ELAVL3	*SMC3*	*NR3C2*	*MYO1E*
TCF4	*SLC1A2*	*AFF2*	*ACTC1*
NF1	*SOX6*	*SKI*	*WDR87*
DYNC1H1	*C12orf57*	*KCNB1*	*PLAC8L1*
PSMD12	*WASF1*	*NACC1*	*ZBTB7A*
CACNA1E	*PPP2CA*	*PCDH19*	*ZBTB18*
SLC9A6	*PCCB*	*KATNAL2*	*H2AC6*
LDB1	*STAG1*	*DSCAM*	*PURA*
CHAMP1	*OCRL*	*BRSK2*	*PLK5*
RORB	*USP9X*	*SATB1*	*SHISA6*
GIGYF2	*HERC2*	*KCNQ3*	*RFX8*
MBD5	*TTI2*	*RELN*	*RYR2*
DDX3X	*UNC13A*	*PAX5*	*EGLN2*
ADSL	*SYNE1*	*SCN8A*	

Supplementary Table 2. List of genes included in the screens

ARX	*TSC1*	*NCOR1*	*POLR3A*
CREBBP	*GABRB3*	*SMAD4*	*PAK1*
KMT2E	*MAP1A*	*SAE1*	*CHD1*
PPP5C	*ANKRD11*	*ILF2*	*XPC*
SPAST	*CTNNB1*	*CNTN4*	*SLC45A1*
NRXN3	*SETD5*	*AMT*	*WDR26*
CUL3	*MECP2*	*NLGN4X*	*ATP1A1*
TRIO	*SIN3A*	*KDM6A*	*RAD21*
AP2S1	*NLGN2*	*ASTN2*	*KIF5C*
ARID1B	*CHD3*	*CNKSR2*	*YWHAG*
NEXMIF	*KIAA0232*	*DLG2*	*PPM1D*
KMT2C	*TANC2*	*DIP2C*	*GATM*
NCKAP1	*CHD7*	*ASAP2*	*HCFC1*
RFX3	*PTEN*	*SPARCL1*	*RSRC1*
SRCAP	*DHCR7*	*GRIA1*	*PCCA*
NCOA1	*NBEA*	*TBC1D31*	*DOLK*
ATRX	*HECTD4*	*CACNA1D*	*NR2F1*
POMGNT1	*CHD2*	*SLC38A10*	*RIMS2*
CNOT3	*HRAS*	*UBN2*	*RPS6KA3*
DPYSL2	*PACS1*	*EMSY*	*PACS2*
WAC	*FOXG1*	*PRICKLE2*	*SGSH*
LZTR1	*DEAF1*	*PLXNB1*	*AP1S2*
EP300	*TBL1XR1*	*GRIK2*	*TRAPPC6B*
CHD8	*PTPN11*	*SLC7A3*	*PRKD1*
ZMYND8	*NRXN1*	*CACNB2*	*ANKS1B*
ADNP	*EHMT1*	*APBB1*	*TET3*
TM9SF4	*SETD2*	*LEO1*	*ARID2*
CELF4	*ZBTB20*	*PRKCB*	*TRRAP*
FMR1	*SRPRA*	*NAV2*	*CEP290*
CORO1A	*MYT1L*	*PHB*	*POU3F3*
CTCF	*ZNF292*	*TRAPPC9*	*DMD*
TSC2	*NLGN3*	*KIAA1586*	*VAMP2*
BCKDK	*ELAVL3*	*TET2*	*KCNMA1*
MED13	*TCF4*	*SCN9A*	*MSL3*
RAI1	*NF1*	*NLGN1*	*PDK2*
PHF12	*DYNC1H1*	*CTNND2*	*TNPO3*

KMT5B	*PSMD12*	*RALGAPB*	*SMARCD1*
NRXN2	*CACNA1E*	*DLGAP1*	*GNAO1*
ALDH5A1	*SLC9A6*	*NUDCD2*	*CABP7*
PPP2R5D	*LDB1*	*GPHN*	*CSNK2A1*
NUP155	*CHAMP1*	*MACROD2*	*NAA10*
TRIM23	*RORB*	*CNTNAP2*	*MAPK3*
UBE3A	*GIGYF2*	*DPP10*	*RAB11A*
FOXP1	*MBD5*	*PAK2*	*PPP2R1A*
HDLBP	*DDX3X*	*PLCB1*	*TNPO2*
SON	*ADSL*	*EP400*	*DNM1*
ASH1L	*GRIN2B*	*ROBO2*	*LARP4B*
KDM5B	*ICA1*	*USP7*	*ENO3*
KMT2A	*JARID2*	*MYO5A*	*EFTUD2*
PHF3	*CSDE1*	*QRICH1*	*NSD2*
IRF2BPL	*ZMYND11*	*CDC42BPB*	*CHD4*
DNMT3A	*CUL7*	*MTOR*	*CERT1*
BCL11A	*SPEN*	*RANBP17*	*HMGXB3*
KANSL1	*PHRF1*	*HMGN1*	*PAPOLG*
GRIA2	*P4HA2*	*PLXNA4*	*SF3B1*
KDM3B	*CLASP1*	*CUX1*	*ODC1*
MED13L	*KCNQ2*	*RALA*	*AGO3*
BAZ2B	*CTTNBP2*	*PAX6*	*ZC4H2*
UPF3B	*OPHN1*	*GABRA3*	*KIF1A*
MBOAT7	*KAT6A*	*TBC1D23*	*PDHA1*
HNRNPH2	*ACHE*	*CAMK2B*	*RRP8*
GNAI1	*SNX5*	*CTNNA2*	*FAM104A*
EIF3G	*RBM27*	*SYT1*	*SYNCRIP*
ARHGEF9	*SUPT16H*	*RORA*	*TRA2B*
TRAF7	*AGO1*	*CAMK2A*	*ABI2*
KDM6B	*BTAF1*	*SMC1A*	*HNRNPD*
VPS13B	*SBF1*	*CLCN4*	*TCF12*
MEIS2	*TNRC6B*	*ACTB*	*ANP32A*
SOX5	*NINL*	*ACTL6B*	*CLTC*
PHF21A	*RAB2A*	*SMARCA2*	*MTF2*
VEZF1	*UBR5*	*MEF2C*	*SUSD4*
SCN2A	*ETFB*	*HUWE1*	*GATAD2B*
STXBP1	*TAF6*	*ALG6*	*UNC80*
SMARCC2	*CEP41*	*CCNK*	*BTF3*
ASXL3	*KDM4C*	*YY1*	*NONO*

RERE	*ZMIZ1*	*NOVA2*	*TMEM178A*
POGZ	*ELP4*	*ATP1A3*	*PRKCA*
SCN1A	*ERBIN*	*RHEB*	*KAT6B*
TBCK	*SEMA5A*	*SMC3*	*KCNJ6*
ANK2	*CCNG1*	*SLC1A2*	*STC1*
GABRB2	*CPEB4*	*SOX6*	*STARD9*
PHIP	*CCT4*	*C12orf57*	*DVL3*
GIGYF1	*SRSF11*	*WASF1*	*RPL26*
TLK2	*APH1A*	*PPP2CA*	*PRPF18*
CASK	*CNR1*	*PCCB*	*SRRM2*
ZNF462	*SET*	*STAG1*	*H1-4*
LRRC4C	*SLC35B1*	*OCRL*	*MAP2K1*
CACNA1C	*LRP1*	*USP9X*	*TMEM42*
ANK3	*SLC12A5*	*HERC2*	*KCND3*
SETBP1	*RPS10P2-AS1*	*TTI2*	*ASXL1*
HNRNPU	*KDM5C*	*UNC13A*	*SNAPC5*
NR4A2	*HIVEP3*	*SYNE1*	*PUF60*
TRIP12	*SMARCA4*	*RLIM*	*SKIDA1*
SLC6A1	*AKAP9*	*DHX30*	*PSMG4*
CACNA2D3	*ZC3H4*	*CDK8*	*PBX1*
DYRK1A	*CHMP1A*	*SNX14*	*ACKR3**
BRAF	*CC2D1A*	*AHI1*	*AGTPBP1**
AUTS2	*PER2*	*RAC1*	*CXCR4**
UBR1	*TERF2*	*GABBR2*	*DLX1**
DIP2A	*MYH10*	*FBXO11*	*DLX2**
TAOK1	*UNC79*	*GALNT2*	*ERBB4**
SHANK2	*AGO4*	*LNPK*	*PAFAH1B1**
WDFY3	*CAPRIN1*	*MED12L*	*ZEB2**
NAA15	*USP15*	*HDAC8*	*NKX2.1**
NIPBL	*DENR*	*TAF1*	*LHX6**
PTCHD1	*ST8SIA2*	*NFIB*	*SOX2**
NSD1	*CDH13*	*NTRK2*	*PAX6**
SNAP25	*FAM200B*	*PPP1CB*	*SOX6**
SLC35A2	*ALG13*	*FAM200A*	
YTHDF3	*SEPTIN10*	*WDR45*	

*Indicate non-NDD genes that have known function in interneuron development

Supplementary Table 3. Candidate genes of the interneuron generation screen

Symbol	casTLE Effect	casTLE Score	Effect size of individual sgRNA				
SMAD4	-2.8	16.7	-3.25	-2.94	-2.39	-1.53	-0.0459
CSDE1	-2.7	15.9	-3.02	-2.83	-2.82	-1.62	-0.109
NAA10	1.9	9.85	-0.762	-0.379	1.16	2.3	2.32
TTI2	1.4	7.8	-1.34	-0.521	-0.0102	1.82	1.87
PPM1D	1.8	7.26	0.223	0.588	0.734	1.69	2.27
SYNCRIP	-2.6	6.74	-3.12	-1.94	-1.1	-0.761	0.712
NSD1	-1.8	6.52	-2.45	-1.78	-1.77	-0.194	1.03
FOXG1	-1.8	6.19	-2.23	-2.12	-1.55	0.575	1.29
ACTB	-2.1	5.02	-2.53	-2.16	-0.667	0.533	1.05
SRSF11	-1.5	4.85	-2.03	-1.67	-1.53	0.473	0.908
POU3F3	-1.8	4.07	-2.35	-1.76	-0.888	0.419	0.442
PPP2R1A	-1.6	2.98	-2.08	-1.8	-0.686	0.192	0.408
RHEB	-1.4	2.42	-1.93	-1.7	-0.43	0.688	1.03

Supplementary Table 4. Candidate genes of the Interneuron migration screen

Symbol	casTLE Effect	casTLE Score	Effect size of individual sgRNA				
EFTUD2	-4.8	7.67	-5.2	-3.24	-1.83	-1.34	-0.0172
STARD9	-4.5	5.2	-4.9	-2.52	-1.95	-0.55	1.36
NR4A2	-4.4	4.94	-4.83	-2.81	-1.81	-0.87	0.191
PRKD1	-4.4	4.27	-4.75	-3.76	-1.16	-1.12	0.773
CHD4	-4.3	8.94	-4.58	-4.32	-2.85	-2.33	-1.22
LNPK	-4	4.92	-4.56	-2.72	-2.45	-1.22	0.742
COL4A3BP	-3.9	1.75	-4.32	-3.07	-0.692	0.791	1.46
SRRM2	-3.8	1.46	-4.18	-3.14	-0.635	0.994	2.45
HRAS	-3.8	1.3	-4.18	-3.07	-0.264	0.372	0.97
DPYSL2	-3.7	1.77	-3.87	-3.85	-1.21	0.821	0.91
CAMK2B	-3.7	1.57	-3.83	-3.81	-0.89	-0.436	0.0177
NCOA1	-3.6	3.47	-4.17	-3.47	-3.24	0.451	0.873
GABRA3	-3.6	1	-3.8	-3.67	0.422	1.3	1.46
AMT	-3.5	3.05	-4.07	-3.43	-2.3	0.167	1.08
HCFC1	-3.5	1.25	-3.97	-3.17	-0.552	-0.337	0.129
GIGYF1	-3.5	1.18	-3.97	-3	-0.452	-0.244	0.92
NLGN1	-3.5	0.99	-3.87	-3.52	0.177	0.756	1.39
RAC1	-3.3	1.89	-3.69	-3.33	-1.57	-0.0948	0.792
SUPT16H	-3.3	1.57	-3.87	-3.23	-1.23	0.282	1.16
ALG6	-3.2	2.17	-3.89	-3.15	-1.76	0.0329	0.516
USP7	-3.1	4.61	-3.35	-3.29	-2.18	-2.02	1.35
TERF2	-3.1	2.29	-3.86	-2.97	-1.83	-0.171	0.847
PHB	-3	1.89	-3.66	-3.01	-1.55	1.67	1.81
NCKAP1	-2.9	4.04	-3.48	-2.95	-2.45	-1.38	1.78
SEMA5A	-2.9	2.55	-3.6	-2.89	-2.02	0.233	1.51
TLK2	-2.8	3.59	-3.18	-2.98	-2.47	-0.694	0.324
KIF5C	-2.7	1.18	-3.14	-2.77	0.37	0.5	1.04
CHD8	-2.6	3.17	-3.24	-2.66	-2.23	-0.364	0.175
ILF2	-2.5	2.6	-3.03	-2.53	-1.38	-0.964	-0.12
ERBB2IP	-2.5	1.66	-2.91	-2.6	-0.653	-0.316	0.454
TRIM23	2.5	6.19	-0.998	-0.973	0.201	2.97	3.06
TCF4	3.1	14.6	-1.05	0.705	2.66	3.55	3.65
RAI1	4.7	6.05	-0.953	-0.0709	0.224	2.86	5.3

Supplementary Table 5. P values and sample size for Fig. 2f and h

Figures	Comparisons	Days	Genes	P value (f) /adjusted P value (h)	Cas9-CTL sample size	KO sample size
Fig.2f	CSDE1 vs Cas9-CTL	8	na	0.1647	25	18
	CSDE1 vs Cas9-CTL	10	na	0.9191	20	30
	CSDE1 vs Cas9-CTL	12	na	0.0559	10	15
	CSDE1 vs Cas9-CTL	25	na	<0.0001	24	26
	CSDE1 vs Cas9-CTL	35	na	0.0001	16	16
	CSDE1 vs Cas9-CTL	40	na	0.0002	15	15
	SMAD4 vs Cas9-CTL	8	na	0.0007	25	29
	SMAD4 vs Cas9-CTL	10	na	0.0002	20	25
	SMAD4 vs Cas9-CTL	12	na	0.0115	10	15
	SMAD4 vs Cas9-CTL	25	na	<0.0001	24	23
	SMAD4 vs Cas9-CTL	35	na	0.0002	16	15
	SMAD4 vs Cas9-CTL	40	na	<0.0001	15	16
Fig.2h	CSDE1 vs Cas9-CTL	na	DLX2	0.00003462	13	12
	CSDE1 vs Cas9-CTL	na	LHX6	4.6152E-06	13	12
	CSDE1 vs Cas9-CTL	na	NKX2.1	0.000006921	13	12
	CSDE1 vs Cas9-CTL	na	SOX6	0.001285714	13	12
	CSDE1 vs Cas9-CTL	na	LHX8	6.9228E-06	13	12
	CSDE1 vs Cas9-CTL	na	NR2F2	0.000666675	13	12
	CSDE1 vs Cas9-CTL	na	DBX1	0.0018	13	12
	CSDE1 vs Cas9-CTL	na	PROX1	0.010164706	13	12
	CSDE1 vs Cas9-CTL	na	NR2F1	0.0006	13	12
	SMAD4 vs Cas9-CTL	na	DLX2	0.000830769	13	13

	SMAD4 vs Cas9-CTL	na	LHX6	0.000654545	13	13
	SMAD4 vs Cas9-CTL	na	NKX2.1	0.00144	13	13
	SMAD4 vs Cas9-CTL	na	SOX6	0.00072	13	13
	SMAD4 vs Cas9-CTL	na	LHX8	6.87343E-05	13	13
	SMAD4 vs Cas9-CTL	na	NR2F2	0.00075	13	13
	SMAD4 vs Cas9-CTL	na	DBX1	0.1129	13	13
	SMAD4 vs Cas9-CTL	na	PROX1	3.11508E-05	13	13
	SMAD4 vs Cas9-CTL	na	NR2F1	6.9228E-06	13	13

Supplementary Table 6. sgRNA sequences used in this study

Experiment	Gene	sgRNA
Generate KO cell pool	*SMAD4*	AUAACAGCUAUAACUACAAA,AAAAAGAGCAAUUGAAAGUU,ACUAUGCACAAUGCUCAGAC
	TERF2	GCUGUUUCUAUCAUGGCCGC,CUGGGUCACGCACGACGCCC,GCGCGCCCUCCCCGCCCUCC
	CSDE1	ACUUUGAUGCGUCCUGGCAA,AGACAAAGAUACGAAAUCCA,CUCGCUCUAAUUUGUCACGU
	SYNCRIP	UUUACCUCAGUGCCAACAGA,UUUACCUCAGUGCCAACAGA,UUUAAUUUACAGAUAUUUGU
	LNPK	UCAAGCAUUGGAAGAAUUUA,UGUAAACAGAUAGAGAACUG,AGCAAAAAAUGGGAGUGUCA
	ATL1	CUGUUCAACAGGUGGAUUUU,GAGGAGCCAGUGAAAAAGGC,UGCAGUUUCAUCUAACUCAA
Examine heterozygous/ homozygous state	*SMAD4 #1*	GAUGUGCCAUAGACAAGG
	SMAD4 #2	GUAUGGUAACACAUUUACU
	MED13L #1	GUAAUGUAUGCACAAGUG
	MED13L #2	GCUAACCUGCUUUGUGUA
	BCKDK #1	GUGCUGAGGAGCGGUCCCGG
	BCKDK #2	GCGAGUGCGGGUCCCAGGA
	CSDE1 #1	GAAACAAGCCCGCUGUCA
	CSDE1 #2	GUCGAAGGGAACGUUCAGC
Mouse slice electroporation	*Lnpk*	AAAAAUAAUACUUACAUCAA,AGUCUUGCUGUAAACUCAUC,AGAUUGCAAAAAUUAUGGGU

Supplementary Table 7. Primers used to amplify genomic DNA for NGS

Primer name	Sequence
LNPK-Forward	ACACTCTTTCCCTACACGACGCTCTTCCGATCTTTTTCTTAAATATCTTTCCTGCTAAAA
TERF2-Forward	ACACTCTTTCCCTACACGACGCTCTTCCGATCTGGAACTACGGCGTCTGAGAA
CSDE1-Forward	ACACTCTTTCCCTACACGACGCTCTTCCGATCTTGAGAAATCTCCAGCTAAAATCG
SYNCRIP-Forward	ACACTCTTTCCCTACACGACGCTCTTCCGATCTCCACCTCCAGATTCCGTTTA
SMAD4-Forward	ACACTCTTTCCCTACACGACGCTCTTCCGATCTTTTCCTTGCAACGTTAGCTG
LNPK-Reverse	GACTGGAGTTCAGACGTGTGCTCTTCCGATCTTCAGAAAAGGAAAAGATCAACAA
TERF2-Reverse	GACTGGAGTTCAGACGTGTGCTCTTCCGATCTCGCCTCGTGGAAGTAGAACT
CSDE1-Reverse	GACTGGAGTTCAGACGTGTGCTCTTCCGATCTAACATTGGCTTTCGCATATT
SYNCRIP-Reverse	GACTGGAGTTCAGACGTGTGCTCTTCCGATCTAGCCTCCTGAGCTGCTTCTT
SMAD4-Reverse	GACTGGAGTTCAGACGTGTGCTCTTCCGATCTAGCCTCCCATCCAATGTTCT

Supplementary Table 8. Primers for RT-qPCR

Gene	Forward primer	Reverse primer
GAPDH	CATGAGAAGTATGACAACAGCCT	AGTCCTTCCACGATACCAAAGT
PROX1	CTGAAGACCTACTTCTCCGACG	GATGGCTTGACGTGCGTACTTC
NR2F2	TGCACGTTGACTCAGCCGAGTA	AAGCACACTGAGACTTTTCCTGC
SOX6	GCCTAAGTGACCGTTTTGGCAG	GGCATCTTTGCTCCAGGTGACA
DBX1	GTTTGGAGTGAACGCCATCCTC	CGAAGTAGGGAAAGGCGAAGGT
DLX2	ACGGGAAGCCAAAGAAAGTC	TTTTGGAAACGCCGCTGAAG
NKX2.1	ACCACAAAGGCCAAACTGCTG	TGAGATTGGATGCGCTTGGTTG
LHX8	TGCTTTGCCTGCTTTTCCTG	TGCAGAGGACTTTCTCTTCCAC
LHX6	TGAATTGTGCGATGCCAACG	TAACATTGGGGGTTGTCAGC
NR2F1	TGCCTCAAAGCCATCGTGCTGT	CAGCAGCAGTTTGCCAAAACGG
LNPK	CAGAAAGGACTGTTACTCCAGCC	GCTAAAGGTGGACCTGGAGGAT
Lnpk	CGCCAGAAAGAACTGTTGCTCC	TATCCAGAGCACCTCGTTCTCG
Gapdh	CATCACTGCCACCCAGAAGACTG	ATGCCAGTGAGCTTCCCGTTCAG
Cas9	Primers were ordered from System Bioscience Cas9-PR-1	

Supplementary Table 9. PCR primers used in this study

Experiment	Gene	Forward primer	Reverse primer
Generate KO cell pool	*LNPK*	TTCCCTGAATCTTTGCCAAT	TGGATTACAAATGAAGGTTTAGAACA
	TERF2	CGCGTCCCTAGCATAGTAGC	TCCCGGATCTGTCTGAAGTC
	CSDE1	TGGTTGACTCGTGTGACCAT	CCTGTCCAGATTTCCATTCAA
	SYNCRIP	TGAACAGGACCCTGGACTTC	CGTGAAAACTGAAGGGGAAA
	SMAD4	CCAGAGCAATTTCATCTTTTCC	CGAAGTGTGGGTTTTCTGCT
	ATL1	TCCTCCTGGGATATAGTGTGAA	TGAAGTGACAGAAGCACAGAAGA
Examine heterozygous/homozygous state	*SMAD4 #1 and #2**	CCAGAGCAATTTCATCTTTTCC	CGAAGTGTGGGTTTTCTGCT
	MED13L #1	TCTTGTGCTAGACACTGAGAC	TTACTGGGCAAGATGGTTATCC
	MED13L #2	ACATACATTCTTCACTGGGAGG	CAGTGGATCTTAGCCTGGTTTC
	BCKDK #1 and #2	TGCGGGTTCTGTGGATTC	CAAGCAATCGTCTGCCACTCTG
	CSDE1 #1	ACAGTCCCAGAGAAAGATGACC	AAAGCCAAATGCCTCCTGAAGC
	CSDE1 #2	GTCTCTGGGCAAATGCTTCCAC	TCTTCCTCTGAGACCCTGCATC

*Same as the primers used in generating KO pool

Supplementary Table 10. Coordinates of CNVs in SNP array

hiPS cell lines	Length (Mb)	CN	Chr:start-stop (hg19)
1205-4	NA	NA	NA
1208-2	0.369509	duplication	chr2:113067622-113437130
	0.116394	deletion	chr8:105069228-105185621
	0.476852	duplication	chr19:27738372-28215223
	0.754378	deletion	chr1:25413466-26167843
2242-1	0.191237	deletion	chr21:14766737-14957973
	0.367091	duplication	chr8:31950827-32317917
SEC61B-mEGFP	0.112539	duplication	chr5:351615-464153
	0.875943	deletion	chr7:69282553-70158495

Supplementary Fig. 1

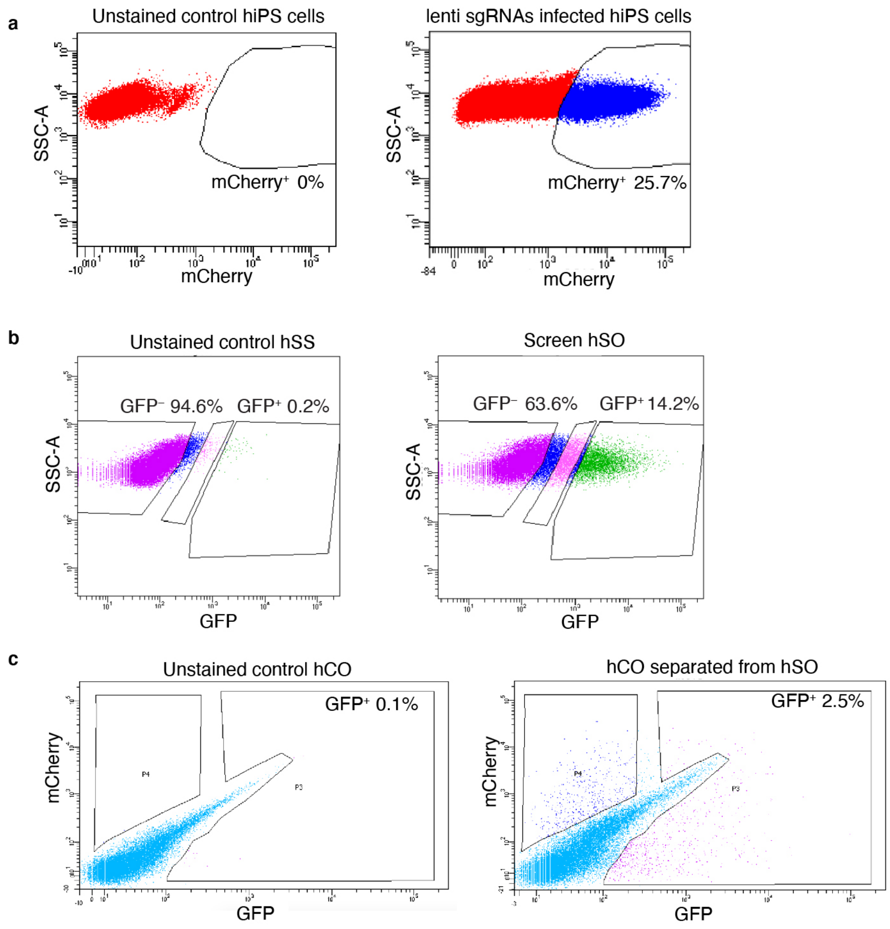

Supplementary Fig. 1. FACS gating strategies. **a**-**c**. Representative FACS gating strategies to sort lentivirus encoding sgRNAs library-infected cells (mCherry$^+$, **a**), eGFP$^+$ and eGFP$^-$ cells from hSO (**b**), and eGFP$^+$ cells from hCO (**c**) as illustrated in Fig 1b. Control hiPS cells, control hSO, and control hCO were used as negative controls.
