# Supplementary material for: Assembloid CRISPR screens reveal impact of disease genes in human neurodevelopment
Source: Nature. 2023 Sep 27;622(7982):359–66. doi: 10.1038/s41586-023-06564-w (PMC10567561; doi:10.1038/s41586-023-06564-w)
Supplement: Supplementary file 2 — Reporting Summary [file 41586_2023_6564_MOESM2_ESM.pdf]

nature portfolio

Corresponding author(s): Sergiu Pasca

Last updated by author(s): July 19, 2023

Reporting Summary

Nature Portfolio wishes to improve the reproducibility of the work that we publish. This form provides structure for consistency and transparency in reporting. For further information on Nature Portfolio policies, see our Editorial Policies and the Editorial Policy Checklist.

Statistics

For all statistical analyses, confirm that the following items are present in the figure legend, table legend, main text, or Methods section.

n/a	Confirmed	
☐	☒	The exact sample size (*n*) for each experimental group/condition, given as a discrete number and unit of measurement
☐	☒	A statement on whether measurements were taken from distinct samples or whether the same sample was measured repeatedly
☐	☒	The statistical test(s) used AND whether they are one- or two-sided<br>*Only common tests should be described solely by name; describe more complex techniques in the Methods section.*
☒	☐	A description of all covariates tested
☐	☒	A description of any assumptions or corrections, such as tests of normality and adjustment for multiple comparisons
☐	☒	A full description of the statistical parameters including central tendency (e.g. means) or other basic estimates (e.g. regression coefficient) AND variation (e.g. standard deviation) or associated estimates of uncertainty (e.g. confidence intervals)
☐	☒	For null hypothesis testing, the test statistic (e.g. *F*, *t*, *r*) with confidence intervals, effect sizes, degrees of freedom and *P* value noted<br>*Give P values as exact values whenever suitable.*
☒	☐	For Bayesian analysis, information on the choice of priors and Markov chain Monte Carlo settings
☒	☐	For hierarchical and complex designs, identification of the appropriate level for tests and full reporting of outcomes
☒	☐	Estimates of effect sizes (e.g. Cohen's *d*, Pearson's *r*), indicating how they were calculated

Our web collection on statistics for biologists contains articles on many of the points above.

Software and code

Policy information about availability of computer code

Data collection	Image Lab (V 6.0.1) was used for gel image acquisition. LAS-X (Leica) software was used for acquiring the microscopy data. Image Studio (V5.2) was used to acquire western blotting data. QuantStudio Real-Time PCR software (V1.7.1) was used to collect qPCR data. NovoExpress (V1.3.0) was used to generate flow cytometry data. Bowtie 1.2.2 was used to aligne sequencing reads.
Data analysis	Flow optometry data were analyzed with FlowJo (V10.8.1). The GraphPad Prism (V9.1.0) was used for statistical analyses. Fiji (ImageJ, V1.0 and V1.53f51, and ImageJ2, V2.3.0/1.53q) was used for image quantification and processing. Imaris (V9.7.0) was used to process images. R 4.1.1 and Python 3.8.5 were used to analyze sequencing data. R version 4.1.2 was used to analyze disease genes expression. casTLE (https://bitbucket.org/dmorgens/castle/downloads/) was used to analyze CRISPR screen data. Image Studio (V5.2) was used to analyze western blotting data. ICE analysis (Synthego) was used to analyze the heterozygous/homozygous state of each mutant clone.

For manuscripts utilizing custom algorithms or software that are central to the research but not yet described in published literature, software must be made available to editors and reviewers. We strongly encourage code deposition in a community repository (e.g. GitHub). See the Nature Portfolio guidelines for submitting code & software for further information.

Data

Policy information about availability of data

All manuscripts must include a data availability statement. This statement should provide the following information, where applicable:

- Accession codes, unique identifiers, or web links for publicly available datasets
- A description of any restrictions on data availability
- For clinical datasets or third party data, please ensure that the statement adheres to our policy

The data that support the findings of this study are available on request from the corresponding author. The following public datasets were used to support this study: the Simons Foundation Autism Research Initiative (SFARI) database (https://gene.sfari.org/). The single-cell RNA-sequencing data from Dlxi1/2b::eGFP+ cells were generated by Birey et al.6 and were downloaded from Gene Expression Omnibus (GEO) under accession numbers GSE93811. Trevino et al.30 data were downloaded from the Gene Expression Omnibus with the accession number GSE162170. Bulk RNA-seq data from the developing human cortex were generated by psychENCODE (BrainSpan, downloaded from: http://development.psychencode.org/).

Human research participants

Policy information about studies involving human research participants and Sex and Gender in Research.

Reporting on sex and gender	N/A
Population characteristics	N/A
Recruitment	N/A
Ethics oversight	N/A

Note that full information on the approval of the study protocol must also be provided in the manuscript.

Field-specific reporting

Please select the one below that is the best fit for your research. If you are not sure, read the appropriate sections before making your selection.

☒ Life sciences ☐ Behavioural & social sciences ☐ Ecological, evolutionary & environmental sciences

For a reference copy of the document with all sections, see nature.com/documents/nr-reporting-summary-flat.pdf

Life sciences study design

All studies must disclose on these points even when the disclosure is negative.

Sample size	Sample sizes were estimated empirically, based on previous studies (Birey et al., Nature 2017; Marton et al., Nature Neuroscience; Pasca et al, Nature Medicine 2019; Yoon et al., Nature Methods 2019; Miura et al., Nature Biotechnology 2020; Han et al., Nature 2019; Birey et al., Cell Stem Cell 2022).
Data exclusions	When analyzing the percentage of saltatory migrating cells that showed ER displacement, 4 images containing densely packed fast-moving cells that are different from other collected images were excluded. In addition, organoids having less than 3 migrating cells were not included.
Replication	Data shown in representative experiments was repeated with similar results in at least 3 independent experiments. All attempts at replication were successful. Sample sizes for other experiments were indicated in Figure legends. Data in Extended Data Fig. 10 e-h was collected from multiple organoids in one differentiation and part of these results verified findings in Fig. 3h and Fig. 4 f-i. NGS validation of the deletion of the candidate genes was performed once as it is standard practice and results were consistent with PCR genotyping experiments.
Randomization	Organoids and assembloids were randomly picked from cell culture plates for each assay. For the experiment used mouse tissues, mouse brain slices were randomly grouped for control and experimental treatment.
Blinding	Blinding of genotypes was used for all imaging analysis, flow cytometry analysis, and organoid size measurements. Blinding was not used or was not relevant for other experiments.

Reporting for specific materials, systems and methods

We require information from authors about some types of materials, experimental systems and methods used in many studies. Here, indicate whether each material, system or method listed is relevant to your study. If you are not sure if a list item applies to your research, read the appropriate section before selecting a response.

Materials & experimental systems

n/a	Involved in the study	
☐	☒	Antibodies
☐	☒	Eukaryotic cell lines
☒	☐	Palaeontology and archaeology
☐	☒	Animals and other organisms
☒	☐	Clinical data
☒	☐	Dual use research of concern

Methods

n/a	Involved in the study	
☒	☐	ChIP-seq
☐	☒	Flow cytometry
☒	☐	MRI-based neuroimaging

Antibodies

Antibodies used	Anti-GFP (Chicken, GeneTex, GTX13970, 1:1000 dilution), Lot # 822104593<br>Anti-GAPDH (mouse, 1:5000, GeneTex,GTX627408), lot#41268<br>Anti-LNPK (rabbit, 1:500, Sigma, HPA014205), lot# 000013636<br>Anti-CSDE-1 (Rabbit, Abcam, ab201688, Lot# GR3352903-3)<br>Anti-SMAD4 (Mouse, Santa Cruz biotechnology, sc-7966, Lot# I2122)<br>Anti-TERF2 (Rabbit, Novus biologicals, NB110-57130SS, Lot# D107745-1)<br>Anti-hnRNP Q (SYNCRIP, mouse, Sigma, 05-1517, Lot# 3485009)<br>Anti-Cleaved Caspase-3 (rabbit, Cell Signaling Technology, 9661, lot# 47)<br>Anti-SOX2 antibody (goat, R&D, AF2018, lot# KOY0622062)<br>Anti-NeuN antibody (mouse, Abcam, ab104224, lot# GR3341933-1)<br>Anti-Atlastin-1 antibody (mouse, Sigma, MABN1831, Clone 3194, lot# 3837025)<br>Goat Anti-Mouse IgG Polyclonal Antibody (IRDye 680RD, 1:10,000, LI-COR Biosciences, 926–68070)<br>Goat Anti-Rabbit IgG Polyclonal Antibody (IRDye 800CW, 1:10,000, LI-COR Biosciences, 926–32211)<br>Alexa Fluor® 488 AffiniPure Donkey Anti-Chicken IgY (Jackson ImmunoResearch 703-545-155)<br>Alexa Fluor Plus 647 donkey anti-goat IgG (H&L) (Thermo Fisher Scientific, A32849)<br>Alexa Fluor 647 donkey anti-mouse IgG (H&L) (Thermo Fisher Scientific, A31571)<br>Alexa Fluor 568 donkey anti-rabbit IgG (H&L) (Thermo Fisher Scientific, A10042)
Validation	Anti-GFP: Used in our previous study (Miura et.al., Nature Biotechnology, 2020) and in 31 studies according to the manufacture's website.<br>Anti-GAPDH: This antibody has been used in at least several hundreds of studies according to the manufacture's website.<br>Anti-LNPK: According to the manufacture's website, this antibody has been validated by the Human Protein Atlas project. In addition, this antibody has also been used by several other studies, including Breuss et.al., The American Journal of Human Genetics, 2018 and Yuniati et.al., Cell Reports, 2020.<br>Anti-CSDE-1: This antibody has been tested on several positive samples according to the manufacture's website.<br>Anti-SMAD4: This antibody has been used in more than 10 studies according to the manufacture's website.<br>Anti-TERF2: This antibody has been used in more than 80 studies according to the manufacture's website.<br>Anti-hnRNP Q: This antibody has been validated with positive samples and have been used by more than 5 studies according to the manufacture's website.<br>Anti-Cleaved Caspase-3: used in our previous study (Pasca et al., Nature Medicine, 2019)<br>Anti-SOX2: this antibody has been used in 186 studies according to the manufacture's website.<br>Anti-NeuN: used in our previous study (Miura et al., Nature Biotechnology, 2020)<br>Anti-Atlastin-1: this antibody has been evaluated by Western Blotting in human brain tissue lysate according to the manufacture's website.<br>Goat Anti-Mouse IgG Polyclonal Antibody: We have used this antibody in our previous study (Birey et.al., Cell Stem Cell, 2022). Based on the manufacture's technical note, this antibody has been validated and used in multiple studies.<br>Goat Anti-Rabbit IgG Polyclonal Antibody: We have used this antibody in our previous study (Birey et.al., Cell Stem Cell, 2022). Based on the manufacture's technical note, this antibody has been validated and used in multiple studies.<br>Alexa Fluor® 488 AffiniPure Donkey Anti-Chicken IgY: this antibody has been used in 261 studies according to the manufacture's website.<br>Alexa Fluor Plus 647 donkey anti-goat IgG (H&L): this antibody has been used in 1010 studies according to the manufacture's website.<br>Alexa Fluor 647 donkey anti-mouse IgG (H&L): this antibody has been used in 1570 studies according to the manufacture's website.<br>Alexa Fluor 568 donkey anti-rabbit IgG (H&L): this antibody has been used in 1025 studies according to the manufacture's website.

Eukaryotic cell lines

Policy information about cell lines and Sex and Gender in Research

Cell line source(s)	Three hiPS cell lines (1205-4, 2242-1, and 1208-2) were reprogrammed at Stanford University from fibroblasts harvested with IRB approval and following written consent.<br>The SEC61B-mEGFP hiPS cell line (AICS-0059 cl.36) was derived at the Allen Institute for Cell Science and the line was obtained under an inter-institutional MTA.<br>HEK293T cell line was ordered from Takara (Cat. # 632180)
Authentication	hiPS cell lines were authenticated by SNP arrays.
Mycoplasma contamination	All cell lines were regularly tested and maintained Mycroplasma free

Commonly misidentified lines (See ICLAC register)	No commonly misidentified lines used in this study

Animals and other research organisms

Policy information about studies involving animals; ARRIVE guidelines recommended for reporting animal research, and Sex and Gender in Research

Laboratory animals	E13-E14 CD1 mouse embryos were used in this study.
Wild animals	No wild animals were used in the study
Reporting on sex	Mouse embryos were used in the experiment without knowing the sex of the embryos.
Field-collected samples	No field collected samples were used in the study.
Ethics oversight	Animal work was performed under protocols approved by the Stanford University's Administrative Panel on Laboratory Animal Care (APLAC) and a University of California, San Francisco Institutional Animal Care and Use Committees.

Note that full information on the approval of the study protocol must also be provided in the manuscript.

Flow Cytometry

Plots

Confirm that:

☒ The axis labels state the marker and fluorochrome used (e.g. CD4-FITC).

☒ The axis scales are clearly visible. Include numbers along axes only for bottom left plot of group (a 'group' is an analysis of identical markers).

☐ All plots are contour plots with outliers or pseudocolor plots.

☒ A numerical value for number of cells or percentage (with statistics) is provided.

Methodology

Sample preparation	Organoids (single organoids cultured in a 96-well plate or up to 8 organoids cultured in a 6-well plate) were incubated with an enzyme solution containing 10U/ml papain (Worthington, LS003127), 1x EBSS (Sigma-Aldrich, E7510-500ML), 0.36% D(+)-Glucose (Sigma-Aldrich), 26 mM NaHCO3 (Sigma-Aldrich), 0.5 mM EDTA (Sigma-Aldrich), DNase (Worthington, LS002007), 6.1 mM L-Cysteine (Sigma-Aldrich), and the ROCK inhibitor Y-27632 (10 µM) at 37°C with 5% CO2 for 15 min; this solution was pre-warmed at 37°C for 30 minutes to activate papain, and then cells were washed with a pre-warmed protease inhibitor solution (1x EBSS, 0.36% D(+)-Glucose, 26 mM NaHCO3, 0.2% trypsin inhibitor (Worthington LS003086)). Organoids were then triturated, and the resulting single cell suspension was centrifuged at 1,200 rpm for 5 minutes. The pellet was resuspended with 3% BSA (Sigma-Aldrich) and then filtered.<br>hiPS cells were dissociated with accutase and filtered before sorting.
Instrument	BD FACS Aria II and ACEA NovoCyte Quanteon 4205.
Software	BD FACSDiva Software for FACS, and NovoExpress software for analyzing the percentage of GFP positive cells. FlowJo was used for data analysis.
Cell population abundance	The purity of the sample were determined by either GFP or mCherry fluorescence.
Gating strategy	In all the sorting and analysis experiments, a similar differentiation or hiPS cells without any fluorescence were used as negative control to set the gate.

☒ Tick this box to confirm that a figure exemplifying the gating strategy is provided in the Supplementary Information.
