# Supplementary material for: Assembloid CRISPR screens reveal impact of disease genes in human neurodevelopment
Source: Nature. 2023 Sep 27;622(7982):359–66. doi: 10.1038/s41586-023-06564-w (PMC10567561; doi:10.1038/s41586-023-06564-w)

Source Data 1: Full western blots for Fig 3j. Dotted areas indicate example lanes used in the figure.

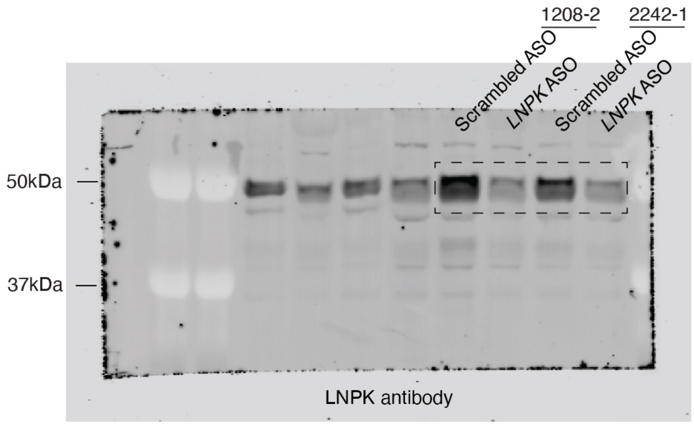

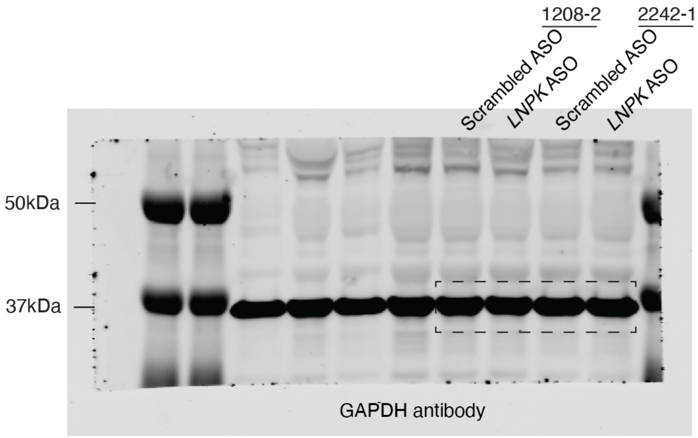

Source Data 2: Full western blots for Extended Data Fig 4. Dotted areas indicate example lanes used in the figure.

Extended data Fig. 4h

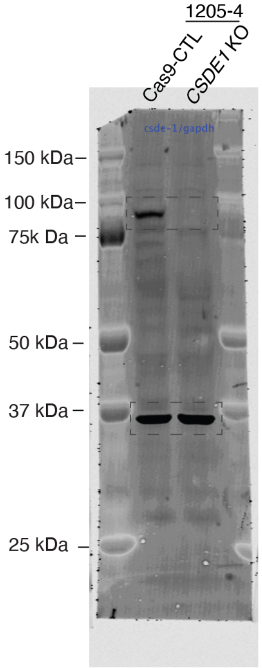

Extended data Fig. 4i

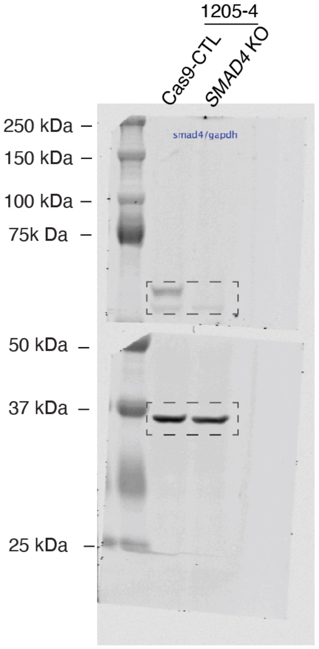

Extended data Fig. 4j

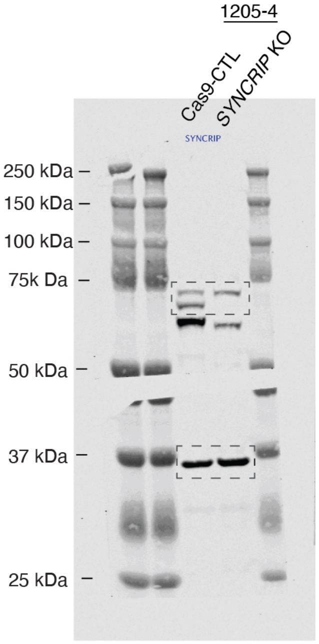

Extended data Fig. 4m

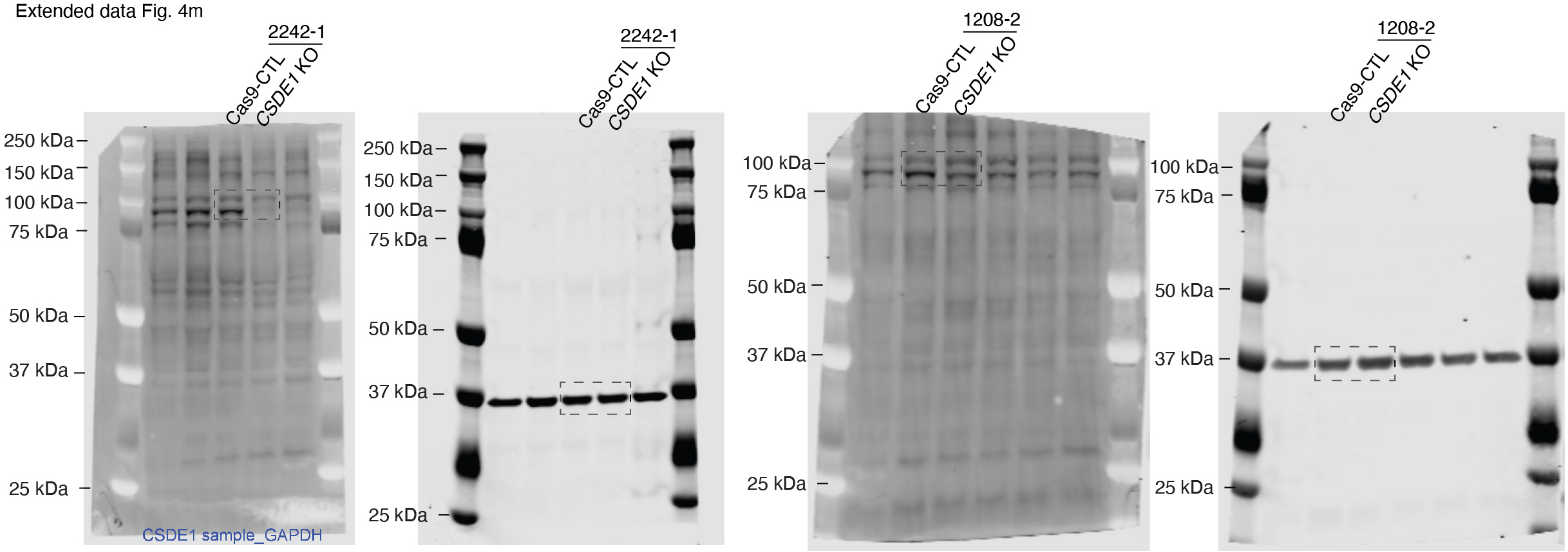

Extended data Fig. 4n

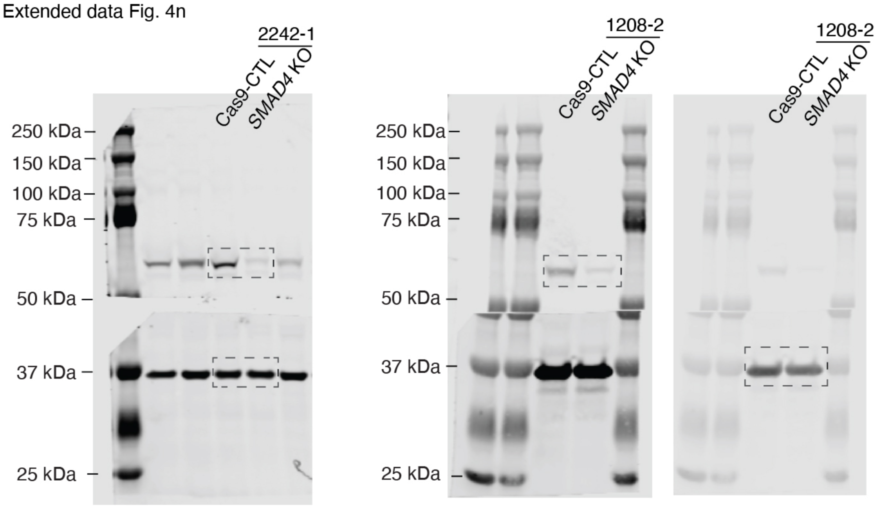

Extended data Fig. 4o

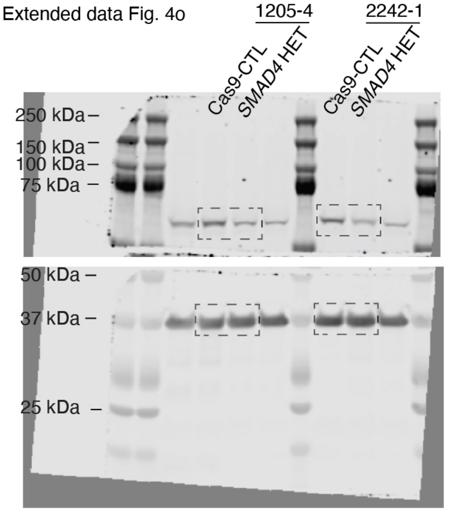

Source Data 3: Full western blots for Extended Data Fig 5f. Dotted areas indicate example lanes used in the figure.

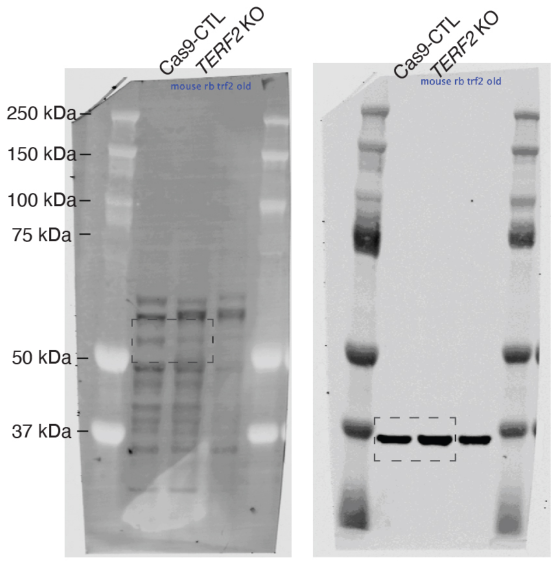

Source Data 4: Full western blots for Extended Data Fig 10j. Dotted areas indicate example lanes used in the figure.

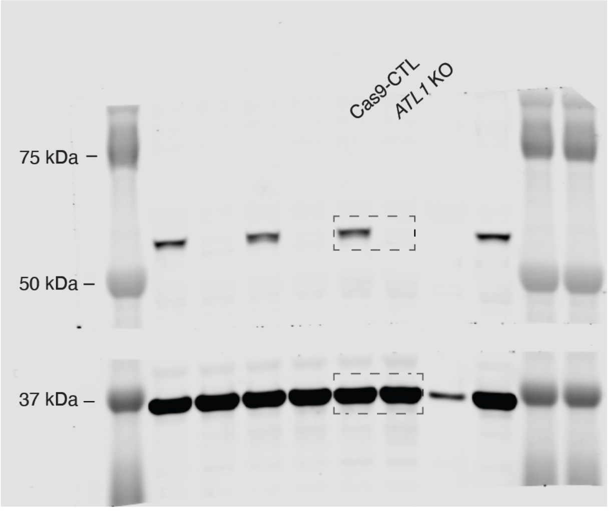

Supplement: Supplementary file 3 — Source Data 1–4. [file 41586_2023_6564_MOESM3_ESM.pdf]
